# Actionable genetic landscape and dynamics in circulating DNA from 12,827 patients with metastatic breast cancer

**DOI:** 10.1038/s41467-026-75976-9

**Published:** 2026-09-22

**Authors:** Ignaty Leshchiner, Arielle J. Medford, Justin Cha, Lesli A. Kiedrowski, Andrzej Niemierko, Jennifer C. Keenan, Charles S. Dai, Laura M. Spring, Beverly Moy, Seth A. Wander, Neelima Vidula, Steven J. Isakoff, Dejan Juric, Mendy Miller, Andrew A. Davis, Lorenzo Gerratana, Ami Shah, Ben H. Park, Adam Brufsky, Joyce O’Shaughnessy, Rebecca J. Nagy, Leif W. Ellisen, Massimo Cristofanilli, Aditya Bardia, Gad Getz

**Affiliations:** 1 Broad Institute of MIT and Harvard, Cambridge, MA USA; 2 Department of Medicine, Boston University, Boston, MA USA; 3 Harvard Medical School, Boston, MA USA; 4 Department of Medicine, Massachusetts General Hospital, Boston, MA USA; 5 Krantz Family Center for Cancer Research, Massachusetts General Hospital, Boston, MA USA; 6 Guardant Health, Palo Alto, CA USA; 7 Department of Radiation Oncology, Massachusetts General Hospital, Boston, MA USA; 8 Washington University in St Louis, St Louis, MO USA; 9 Department of Medicine, University of Udine, Udine, Italy; 10 Department of Medical Oncology, CRO di Aviano, National Cancer Institute, IRCCS, Aviano, Italy; 11 Northwestern University, Chicago, IL USA; 12 Vanderbilt-Ingram Cancer Center, Nashville, TN USA; 13 University of Pittsburgh, Pittsburgh, PA USA; 14 Baylor University Medical Center, Texas Oncology, Dallas, TX USA; 15 Weill Cornell Medicine, New York, NY USA; 16 UCLA Jonsson Comprehensive Cancer Center, Los Angeles, CA USA; 17 Department of Pathology, Massachusetts General Hospital, Boston, MA USA

**Keywords:** Breast cancer, Cancer genetics, Next-generation sequencing, Computational biology and bioinformatics

## Abstract

Plasma cell-free DNA (cfDNA) is a sensitive, noninvasive tool for monitoring the genomic landscape, heterogeneity, and evolution of cancer, aggregating genetic information from multiple metastatic sites. Here, we study 15,564 cfDNA samples from 12,827 patients with metastatic breast cancer (MBC). We classify samples, deep-sequenced with Guardant360, into MBC subtypes using our trained random forest model. Most patients have variants associated with therapeutic resistance and targetability of approved and experimental therapies, including in *PIK3CA, ESR1, PTEN*, and *AKT1*. Frequencies of multiple actionable alterations differed from primary disease, and multiple variants per gene suggest independent resistant clones. The large cohort size enables uncovering clonal co-behaviors, including co-existence and exclusivity of alleles and correlation of allele fractions. cfDNA testing is now recommended in multiple advanced malignancies, including eligibility assessment for clinical trials. We envision this large collection of MBC cfDNA data as an invaluable resource for the design and planning of future clinical trials.

## Abstract (web summary)

Metastatic breast cancer remains a heterogenous disease which is difficult to treat effectively. Here, the authors survey over 15,000 samples via cfDNA analysis and characterize the landscape of targetable alterations across major breast cancer subtypes.

## Introduction

Despite numerous therapeutic advances, breast cancer remains one of the leading causes of cancer death^1^. While early detection confers a favorable prognosis, many patients still develop metastatic disease, at which point patients receive supportive rather than curative therapies, which require continuous adjustment of therapy as cancer progresses. Mutations conferring therapeutic resistance are frequently acquired in metastatic disease^2–4^, and while the genomics of primary breast cancers have been well described, there remains much to learn about metastatic breast cancer (MBC)^5–12^. Our understanding of the genomics of MBC has relied primarily on tissue biopsies, which assess only a single site of the disease and may fail to capture tumor heterogeneity. Furthermore, patients with MBC are often treated with multiple successive lines of therapy over many years; therefore, baseline tissue biopsies will not reflect acquired mutations.

Cell-free DNA (cfDNA) overcomes these issues by aggregating DNA from multiple metastatic sites throughout the body, thus enabling sampling from remote lesions, even in the setting of distant metastatic spread^13^. Collecting cfDNA longitudinally enables noninvasive monitoring of clonal dynamics and changes in the mutational landscape of the progressing tumor, as well as detection of resistant cancer cell populations. Patients with progressing breast cancer typically have circulating tumor DNA in their cfDNA, the levels of which are generally increased in the metastatic setting when compared to primary breast cancer^14^. The Guardant360® assay performed deep targeted sequencing of up to 74 clinically significant genes in cfDNA and has been shown to reach 95% sensitivity in the setting of progressive, previously treated MBC^15^. In contrast to whole-genome or whole-exome sequencing, targeted sequencing evaluates clinically relevant mutations at a high sensitivity even with minimal contribution of tumor DNA to the cfDNA^13^. This high sensitivity is a key advantage of such assay in the context of studying cancer evolution, as new tumor subclones may initially be represented at very low abundances in cfDNA.

In clinical practice, cfDNA analysis is performed typically in one of several contexts: prior to starting a new therapy, at the time of progression, or while on therapy. Plasma-based assays can help with routine selection of genotype-matched targeted therapy, which are associated with improved clinical outcomes^16^. Multiple genotype-targeted agents are in clinical development, and detecting targetable genomic alterations is also critical for clinical trial candidacy. Additionally, treatments exert selective pressure that often leads to the growth of resistant clones, and early identification of resistance could inform treatment decisions and avoid committing patients to ineffective therapies^17–19^. Thus, genomic profiling is part of MBC standard of care and the American Society of Clinical Oncology (ASCO) advises genomic testing, either via tissue or cfDNA testing, in essentially all advanced or metastatic solid tumors to identify potential biomarkers to guide therapy^20,21^. Furthermore, in breast cancer, the approval of treatments like the oral selective estrogen receptor degrader (SERD) elacestrant for *ESR1*^mut^ ER+/HER2- breast cancer has led to ASCO guidelines recommending cfDNA analysis at the time of MBC progression, given *ESR1* mutations are almost always acquired under selective therapeutic pressure; thus, it is likely that serial cfDNA testing will be increasing for MBC^18,19,22^.

Most large publicly available genomic datasets to date either characterize primary tumors, e.g., the Cancer Genome Atlas (TCGA) and the Molecular Taxonomy of Breast Cancer International Consortium (METABRIC)^23^, or tissue biopsy profiling in advanced cancers, e.g., MSK-IMPACT (1918 metastatic tissue samples from 1756 patients), AACR GENIE (6731 metastatic tissue samples from 6131 patients, 856 breast cancer)^24–27^. Other non-public datasets have reported cfDNA-based findings on tumor evolution and resistance in breast cancer and report unique genomic landscapes in the metastatic setting^24,28^. Here we present the analysis of sequencing data from the largest cfDNA-based cohort to date, which includes 12,827 patients with metastatic breast cancer treated with both standard of care and novel therapies; among which 11,456 had detectable variants. Moreover, this cohort profiles a large number of longitudinally collected cfDNA samples (*n* = 1315 patients with variants detected amongst 1470 that underwent serial testing). Overall, we hope that this dataset and analytical framework will serve as a valuable resource for planning future clinical trials, drug development, and biological research in MBC.

## Results

**Fig. 1:**
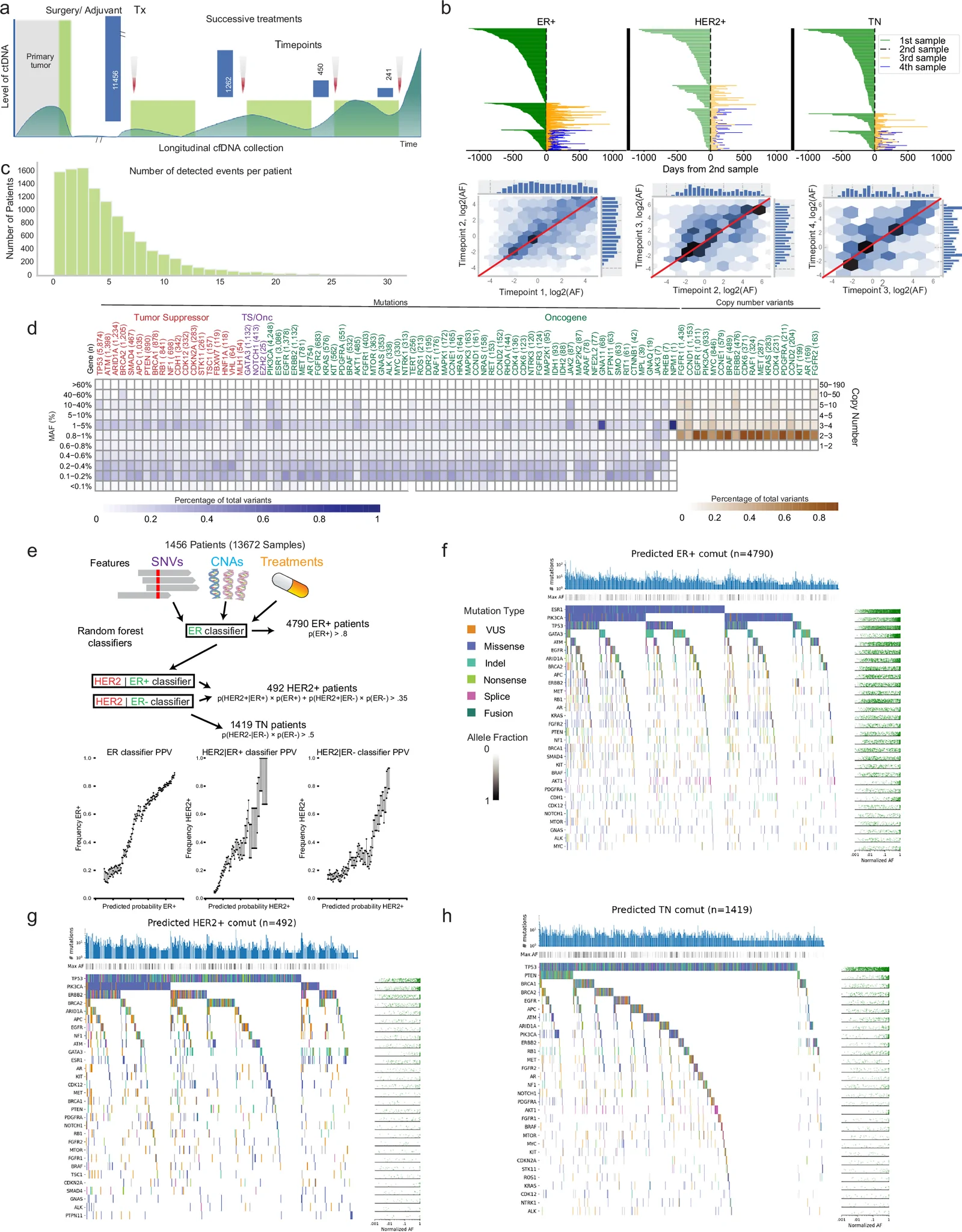
Genomic landscape of cell-free DNA from 12,827 MBC patients on Guardant360 assay. **a** Schematic representation of cfDNA collection during disease progression. **b** Plot of time between 1st and 2nd, 2nd and 3rd, and 3rd and 4th samples stratified by predicted receptor status (top). Dimensional heatmaps of inferred cfDNA purities between 1^st^ −2^nd^, 2^nd^−3^rd^ and 3^rd^−4^th^ timepoints (bottom). **c** Histogram of number of detected events in each patient. **d** Heatmap showing the number, percentage, and proportion of mutations and copy number alterations identified in the 1st Guardant draw for each of the patients with detectable non-synonymous cfDNA alterations. Variants of uncertain significance (VUS) are excluded from this figure. Tumor suppressor and oncogene classifications are based off classifications within the OncoKB database^34^. TS Tumor Suppressor, Onc Oncogene, TS/Onc tumor suppressor or oncogene depending on context (TS, Onc, and TS/Onc determined by OncoKB annotations), MAF mutant allele frequency. **e** Algorithm schema for determining breast cancer receptor-based subtypes based on a random forest classifier using point mutations, copy number amplifications, and treatment information as features. Patients were first classified based on the presence or absence of the estrogen receptor (ER), and then further subdivided based on the presence or absence of amplification of the human epidermal growth factor receptor 2 (HER2). **f** Of the 4930 patients classified as ER+ , there were 273 patients with curated annotations documenting ER+ subtype, and 4657 were predicted to be ER+ based on the cfDNA-based classifier. Each column represents an ER+ patient. The total numbers of mutations detected in each patient are graphed in the top row. Maximum allele frequency (AF) is represented by darkness in the row immediately below. Mutations are documented as lines next to their corresponding gene, with color classification based on type of mutation as shown in the legend on the left. Scatter plots on the right represent the density of normalized AF (AF/max patient AF) meant to approximate cancer cell fraction for point mutations of each gene. **g** Similar to (**e**) for 499 patients (50 annotated, 449 predicted) classified as HER2+ . **h** Similar to (**e**) for 1474 patients (91 annotated, 1383 predicted) classified as triple negative breast cancer (TNBC).

Here we present a cohort of 15,564 samples from 12,827 de-identified patients with MBC with alterations detected via the Guardant 360® cfDNA assay, which analyzes 74 clinically relevant genes (Suppl. Data 1 and 2). There were 13,672 samples (88%), from 11,456 (89%) patients, where at least one alteration was reported, including 48,520 single nucleotide variants (SNV; 44,957 of which were nonsynonymous), 6,066 short insertions or deletions (indels), 10,233 gains, and 56 fusions (Suppl. Data 2). These cfDNA samples were collected as part of standard of care clinical testing, thus testing intervals were determined at the discretion of the ordering provider and varied per individual patient. For reference, guidelines today advise NGS testing at metastatic diagnosis and at treatment progression, though patients may also have undergone testing while on treatment for on-treatment molecular monitoring. Demographic data available through the testing platform included patient-reported gender and age; the vast majority of patients were female (*n* = 11,335, 99%), and mean age was 61 (median 61, range 18–101). Amongst patients with detectable variants, serial cfDNA sampling had been performed in 1470 patients (11%), which included 11,313 patients with detectable alterations at the first time point, 1262 patients with detectable alterations at the second time point and 450 with detectable alterations in the third (or later) timepoint (Fig. 1a). Clinical annotation for breast cancer subtype was available for 285 patients (~3%); positive or negative estrogen receptor (ER) expression and positive or negative human epidermal growth factor receptor 2 (HER2) status were based on ASCO/CAP guidelines. Negative ER and HER2 statuses were used to assume triple negative breast cancer (TNBC) status. Of note, progesterone receptor (PR) is the third receptor assessed in the TNBC definition, however, expression of PR in the absence of ER expression is rare, thus ER served as a surrogate indicator of expression for both hormone receptors (Suppl. Data 2, 3).

To compare the relative abundances and detectability of the tumor DNA in the cfDNA at the initial and later time points, we used as a proxy for purity the highest allele fraction (AF) among the detected somatic mutations (in case of a heterozygous mutation in a diploid tumor, this corresponds to half the tumor DNA component in the cfDNA). Among patients with detectable variants at the initial time points, the highest AF mutation had a median of 2.9% (with an interquartile range (IQR) [0.58%, 13.3%]), while the median highest AF in the second sample was 2.7% (IQR [0.54%, 13.6%]) and 2.6% (IQR [0.54%, 13.9%]) in the third sample. The number of detected variants per patient had a wide range from 0 to 202 but with the majority having ≤5 variants per patient (median of 4, IQR [2,6]) (Fig. 1b). The genomic panel used in this study, like most commercially available tests in this clinical setting, was a targeted panel designed to include oncogenes and tumor suppressor genes commonly mutated across multiple solid tumors or for which rare mutation events were potentially targetable with approved or investigational agents. Of note, this assay cannot fully distinguish somatic alterations from germline variants or from variants resulting from clonal hematopoiesis (CH).

The high sensitivity of the assay allowed detection of multiple alterations even under 1% AF (62.3% of all alterations in the first sampling) (Fig. 1c). The most frequently detected variants across all patients, after consolidating all serial samples (i.e., if a variant was found at any point in serial sampling it was counted for a patient), were in the *TP53* tumor suppressor (*n* = 6078 [47.4%]) and the oncogenes *PIK3CA* and *ESR1* (*n* = 4389 [34.2%] and 3256 [25.4%], respectively), and the most common copy-number gains were detected in *FGFR1* and *CCND1* (*n* = 1530 and 1246, respectively; Fig. 1c). Of note, in some instances, focal amplifications cannot be distinguished from arm level aneuploidy. Assessing copy-number alterations from cfDNA, and in particular when the tumor fraction is low, is not trivial. Nevertheless, 11% (*n* = 1451) of patients had at least one gene estimated to be amplified to more than 4 copies (Fig. 1c).

### cfDNA-based classifier predicts breast cancer subtype

Given the fact that over 90% of patients did not have curated annotations identifying the patients’ breast cancer subtype, which is needed to study the genomic profile of each of the three major breast cancer subtypes, we utilized the known subtype in ~3% of cases, the differences in their patterns of genomic alterations (non-synonymous mutations and amplifications) and available treatment annotations to develop a classifier to predict the tumor subtype. The final classification was based on three Random Forest classifiers that were applied hierarchically. We first predicted whether the sample was ER+ or not, and then predicted the HER2 status with one of two classifiers, each trained separately on ER+ or ER- cases (Fig. 1e; Suppl. Data 4). The classifier was able to confidently classify the majority of tumors to their subtypes, which we evaluated using five-fold cross validation (Fig. 1e). About two-thirds of the samples (*n* = 6903) were confidently assigned (confidence > 75%) to either the ER+ , HER2+ or TNBC subtypes (Fig. 1f–h). In addition to the characteristic genomic events, the classifier used available treatment information (palbociclib, ribociclib, fulvestrant, tamoxifen, abemaciclib, letrozole, exemestane, and anastrozole with ER+ breast cancer; and the use of trastuzumab, pertuzumab, T-DM1, and neratinib with HER+ status) as expected. Since there are double-positive cases (ER+/HER2+), we did not want the classifier to use the HER2 status to predict the ER status. We, therefore, removed the variables related to HER2+ cancer (ERBB2-amplification and anti-HER2+ therapy) when training the ER+ vs ER- classifier. Thus, patients were independently assessed for ER+ and HER2+ status, as performed in clinical practice.

The classification positive predictive value (PPV), assessed by cross-validation, was 88% (120/136) for ER+ cases, 97% (44/45) for HER2+, and 73% (45/62) of predicted ER-/HER2- cases. Cross-validation showed a false positive rate of 12% (16/136), 2% (1/45), and 27% (17/62) for ER+ , HER2+ , and TNBC, respectively. The predicted ER+ subtype was characterized by the most frequent mutations: *ESR1* (58%), *PIK3CA* (54%), *TP53* (33%), *GATA3* (18%), and *ATM* (15%), with other variants having a prevalence lower than 10% (Fig. 1f). Notably, the top two mutations were *ESR1* and *PIK3CA*, which have approved targeted therapies in ER+/HER2- breast cancer and thus indicate the use of genomic targeted therapies as a standard of care^29,30^.

We further validated the classifier by running it on the METABRIC cancer database, with PPVs of 90% (683/758) for ER+ , 85% (245/285) for HER2+ , and 70% (114/161) for TNBC (Suppl. Fig. 1a). In order to assess the predictive power of each individual gene, we also trained 74 separate classifiers, each one with a single gene’s point mutations removed from the input data, and quantified the numbers of patients with predicted receptor status labels switched for each (Suppl. Fig. 1b). The most predictive genes for the ER classification were ESR1 with 3.9% of patients classified as ER+ switching to ER- in the heldout set and TP53 with 2.8% of patients classified as ER- switching to ER+. For HER2 classification, no individual genes had ≥1% of patients switching labels. For TNBC classification, ESR1 was again the most predictive gene with 1.7% of patients switching to TN.

### Frequency of actionable alterations in metastatic breast cancer varies by subtype

The landscape of actionable mutations is constantly changing, and given that not all “actionable” alterations have the same level of clinical significance, we utilized the validated ESMO Scale for Clinically Actionable Molecular Targets (ESCAT) criteria to determine variant actionability. ESCAT is a five-tiered system, where ESCAT I and II mutations describe alterations against which targeted therapies have proven effective in clinical trials^31,32^. The concept of evaluating targetability and actionability is critical to designing genomic-based clinical trials, where investigators must understand the prevalence of genomic targets as well as the frequency of other concurrent targetable alterations and potential resistance mechanisms, in order to define patient populations and estimate accrual. Moreover, recently it has been shown that selecting patients for targeted clinical trials based on cfDNA tests is similarly effective as trials using tissue biopsies^29,33^. We used the full list of confidently classified patients as well as the OncoKB annotated genomic variant database (https://github.com/oncokb/oncokb-annotator) to annotate the variants based on their clinical actionability and matched these to their corresponding ESCAT classification^34^.

**Fig. 2:**
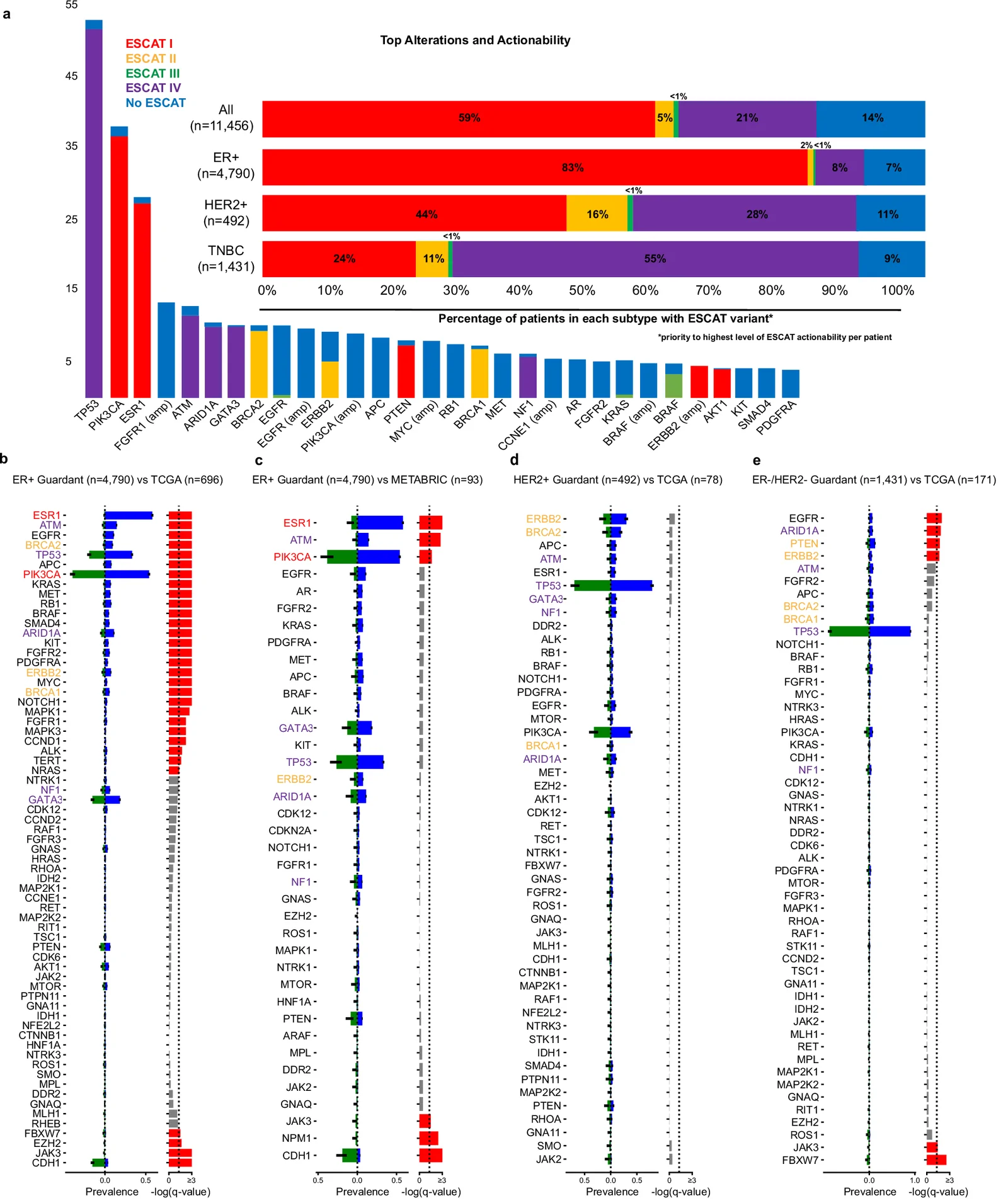
Actionable alterations by the ESMO Scale for Clinically Actionable Molecular Targets (ESCAT). **a** The total number of patients with each gene alteration are graphed against the total number of patients with detectable cfDNA alterations in the cohort (*n* = 11,456 of 12,827) for a percentage. The results of the serial sampling are condensed into each individual patient datapoint, thus any detection of an alteration at any time point is assigned to that patient. Priority is given to the highest ESCAT classification per patient. The ESCAT classification is color coded. Of note, the Guardant360 assay does not completely distinguish germline mutations from somatic, nor does it distinguish clonal hematopoiesis of indeterminate potential (CHIP). *TP53*, *ATM, IDH1* and *IDH2* are genes associated with CHIP. *ESCAT actionability varies by subtype. The color coding in the total patient cohort is reflective of the largest percentage of patients in that ESCAT category (ER+). The subtype-specific color coding is specific to that subtype. For *BRCA1* and *BRCA2* alterations in the subtype figure, a VAF cutoff of 40% was used to presume somatic (ESCAT II) below the cutoff vs germline (ESCAT I in HER2- breast cancer) above the cutoff. **b–e** The frequency of each gene mutation is shown compared to genes described in The Cancer Genome Atlas (TCGA) and the Molecular Taxonomy of Breast Cancer International Consortium (METABRIC) databases. The mutation rate for each gene is represented by the proportion of patients with at least one non-synonymous mutation in that gene. A false discovery rate (q) of less 0.05 is deemed statistically significant and highlighted in red. Error bars are defined using 95% Clopper-Pearson confidence intervals.

Overall, 64% of patients had ESCAT I/II alterations in at least one of their samples (Fig. 2a, Table 1). When classifying by subtype, we identified ESCAT I/II actionable alterations in 85% of patients with predicted ER+ MBC, 60% with predicted HER2+ breast cancer (excluding *ERBB2* amplification), and 35% with predicted TNBC (Fig. 2a).

**Table 1:** Actionable genomic variants in metastatic breast cancer detected via cfDNA (*n* = 11,456 patients with detectable cfDNA)

ESCAT I (target approved for routine clinical care)						
Gene	ESCAT variant (*n*)	ESCAT variant (%)	Non ESCAT variant (*n*)	Non ESCAT variant (%)	All variants (*n*)	Genome-specific targeted therapy
*PIK3CA*	4113	35.9%	240	2.1%	4353	Alpelisib
*ESR1*	3034	26.5%	184	1.6%	3218	Elacestrant
*ERBB2 (amp)*	512	4.5%	0	0.0%	512	Trastuzumab, Pertuzumab, Trastuzumab emtansine, Trastuzumab deruxtecan, Neratinib, Lapatinib, Tucatinib)
*gBRCA2	222	1.9%	0	0.0%	948	Olaparib, Talazoparib
*gBRCA1	115	1.0%	15	0.1%	699	Olaparib, Talazoparib
*NTRK (fusion)*	6	0.1%	25	0.2%	6	Larotrectinib, Entrectinib
*PTEN*	720	6.3%	201	1.8%	921	Capivasertib
*AKT1*	419	3.7%	67	0.6%	486	Capivasertib
*BRAF (V600E)*	88	0.8%	435	3.8%	523	Dabrafenib, Encorafenib, Vemurafenib
*RET (fusion)*	18	0.2%	86	0.8%	104	Selpercatinib, Pralsetinib
ESCAT II (clinical benefit, magnitude of which to be determined)						
Gene	ESCAT variant (n)	ESCAT variant (%)	Non ESCAT variant (n)	Non ESCAT variant (%)	All variants (n)	Genome-specific targeted therapy
*ERBB2 (mut)*	585	5.1%	472	4.1%	1057	Neratinib
*sBRCA2	281	4.0%	716	6.3%	948	Olaparib
*sBRCA1	186	2.5%	938	8.2%	699	Olaparib
ESCAT III (clinical benefit in other tumor types)						
Gene	ESCAT variant (n)	ESCAT variant (%)	Non ESCAT variant (n)	Non ESCAT variant (%)	All variants (n)	Genome-specific targeted therapy
*IDH2*	6	0.1%	78	0.7%	84	Enasidenib
*EGFR*	38	0.3%	1123	9.8%	1161	Erlotinib, Afatinib, Gefitinib, Osimertinib, Dacomitinib
*IDH1*	46	0.4%	52	0.5%	98	Ivosidenib, Olutasidenib
*KRAS (G12C)*	45	0.4%	556	4.9%	601	Sotorasib, Adagrasib
*KIT*	17	0.1%	466	4.1%	483	Imatinib, Sunitinib, Regorafenib, Ripretinib
*PDGFRA*	13	0.1%	454	4.0%	467	Avapritinib
*ALK (fusion)*	7	0.1%	292	2.5%	299	Crizotinib, Lorlatinib, Ceritinib, Alectinib, Brigatinib
*FGFR3 (fusion)*	6	0.1%	64	0.6%	70	Erdafitinib
ESCAT IV (preclinical evidence of actionability)						
Gene	ESCAT variant (n)	ESCAT variant (%)	Non ESCAT variant (n)	Non ESCAT variant (%)	All	Genome-specific targeted therapy
*TP53*	5711	49.9%	348	3.0%	6059	APR-246
*ARID1A*	676	5.9%	530	4.6%	1206	anti-EZH2
*ATM*	603	5.3%	868	7.6%	1471	anti-PARP, anti-ATR
*GATA3*	904	7.9%	261	2.3%	1165	anti-MDM2
*NF1*	333	2.9%	373	3.3%	706	anti-MEK

*germline BRCA1/2 mutation annotated if allele frequency ≥ 40%, somatic BRCA1/2 if < 40%.

The genes with most common ESCAT I/II mutations were *PIK3CA*, *ESR1*, *ERBB2*, *PTEN, BRCA2*, *AKT1*, and *BRCA1*. Here, we emphasize the fact that most approved targeted therapies for MBC are approved in ER+/HER2- breast cancer (*PIK3CA*, *ESR1*, *PTEN, AKT1*). Of note, the assay does not fully distinguish germline from somatic *BRCA1/2* variants. Furthermore, given clinical evidence of actionability of both germline and somatic *BRCA1/2* alterations in breast cancer, these were classified as ESCAT I (germline) and ESCAT II (somatic)^35–37^. ESCAT III alterations were determined as variants that had ESCAT I evidence of actionability for a non-breast cancer indication^34^. The frequency of ESCAT III was relatively low, which highlights the need to study treatment options based on these alterations in genomic basket trials (Table 1, Fig. 2a). This analysis was also performed in each subtype separately, further highlighting the difference in approved targeted therapies between ER+/HER- and the other subtypes (Suppl. Fig. 2).

### Targeted sequencing identifies high frequency of resistance mutations in ER+ metastatic breast cancer

Resistance mutations may emerge under selective pressure, and early identification of these has the potential to influence treatment decisions^32^. We evaluated our ER+ cohort for genomic alterations associated with resistance or reduced response to each of these classes of therapies^38–44^ (8 mutated genes, 7 amplifications; Table 2). The majority of patients (4049/4790; 84%) had one or more events associated with inferior response to established therapies, including 2632 (56%) with oncogenic or likely oncogenic *ESR1* mutations and 2478 (53%) patients with *PIK3CA* mutations. Of note, *ESR1* mutations are strongly predictive of ER positivity, given these are mutations provoked by prior aromatase inhibitor therapy, which is given only in the ER+ setting, thus, the classifier is likely selecting for this subgroup.

**Table 2:** Nonsynonymous genomic alterations in genes associated with resistance to ER+ MBC therapies

Mutations	Resistance	ER+ Guardant (%)	TCGA	*q*-value	METABRIC	*q*-value
*AKT1*	Aromatase inhibitors	4.52%	3.80%	0.52	N/A	N/A
	CDK4/6 inhibitors					
	HER2-targeted therapy					
	SERD					
*ARID1A*	SERD	11.10%	4.50%	0.66	8.60%	0.66
*ERBB2*	SERDs,	7.56%	3.00%	5.2 × 10^−^^6^	4.30%	0.53
	Aromatase inhibitors,					
	*HER2-targeted therapies (trastuzumab, lapatinib)					
*ESR1*	Aromatase inhibitors	57.10%	0.96%	5.1 × 10^−^^225^	7.50%	2.5 × 10^−^^22^
*NF1*	Aromatase inhibitors	6.37%	3.96%	0.023	4.30%	0.66
	SERDs					
*PIK3CA*	Aromatase inhibitors	53.90%	40%	8.8 × 10^−^^12^	39%	0.025
	HER2-targeted therapy					
	SERD					
*PTEN*	PIK3CA	6.45%	5.50%	0.42	8.60%	0.6
	CDK inhibitors					
	SERD					
	Aromatase inhibitors					
*RB1*	CDK4/6 therapy	7.62%	1.60%	4.5 × 10^−^^11^	N/A	N/A
Amplifications	Resistance					
*CCNE1*	CDK4/6 inhibitors	1.10%				
*CDK6*	CDK inhibitors	2.10%				
*EGFR*	Aromatase inhibitors	8.16%				
	SERDs					
*ERBB2*	Aromatase inhibitors	2.65%				
	SERD					
*FGFR1*	SERD	14.90%				
	Aromatase inhibitors					
	CDK4/6 inhibitors					
	HER2-targeted therapy					
*FGFR2*	CDK4/6 inhibitors	0.69%				
	HER2-targeted therapy					
*MYC*	Aromatase inhibitors	5.30%				
	SERD					

*ERBB2 mutations may confer sensitivity to other HER2-targeted therapies (e.g. neratinib).

Interestingly, patients with ER+/HER2- MBC and *ESR1* mutations were recently shown to also have improved responses to the oral SERD elacestrant, recently approved for use in this patient subpopulation. Amongst 2707 patients with ER+/HER2- MBC with any type of *ESR1* mutations, 2683 (99%) harbored mutations in the ligand binding domain, which qualifies patients to receive elacestrant. In addition, we observed that 137 (39%) out of 350 ER+/HER2- patients with serial sampling had newly discovered *ESR1* mutations not identified on initial cfDNA testing. This is consistent with the recent ASCO guidelines that recommend performing ESR1 mutation detection in post-progression samples, with a preference for cfDNA assays, in order to identify patients eligible for elacestrant^22^.

Next, to discover resistance and late driver events, we compared the prevalence of mutations in our cfDNA cohort of progressive and advanced disease with the primary cancer databases, TCGA and METABRIC^45^. We identified targetable genomic alterations and acquired resistance mechanisms at significantly increased frequencies in 31 genes in our ER+ cohort compared to the 696 TCGA ER+ breast cancers (FDR, *q* < 0.05), including *ESR1* (57% vs 1%, *q* = 5.1 × 10^−^^225^), *ERBB2* (7% vs. 3%, *q* = 5.1 × 10^−^^6^), *ARID1A* (11.1% vs. 4.5%, *q* = 1.1 ×10^−^^8^), *NF1* (6.3% vs 4%, *q* = 0.02), and *RB1* (7.6% vs. 1.6%, *q* = 4.5 × 10^−^^11^) (Fig. 2b, Table 2). We found similar results when comparing to METABRIC (Fig. 2c).

The HER2+ predicted cohort had significant or near-significant enrichment of mutations only in *ERBB2* compared to TCGA (*q* = 0.09), and *ERBB2* and *TP53* compared to METABRIC (*q *= 0.01 and 0.07, respectively; Fig. 2d, Suppl. Figs. 3, 4). Finally, the predicted triple negative breast cancers had increased frequency of mutations in *EGFR*, *ARID1A, PTEN*, and *ERBB2* compared to TCGA (*q* < 0.05; Fig. 2e). Of note, since these observations are based on our predicted subtypes, the true subtype-specific frequencies may vary due to classification inaccuracies. While the HER2+ and TNBC predicted cohorts were comparatively smaller than our ER+ predicted cohort, the absolute patient number was still large, with the combined predicted and curated cohort at 492 HER2+ patients and 1,431 TNBC patients. The larger number of acquired mutations in advanced ER+ breast cancer argues for either a more genetically dynamic disease or more effective targeted therapies that elicit specific genetic resistance mechanisms.

### Co-occurrence of cis- and trans-mutations and likely interaction among common alleles

**Fig. 3:**
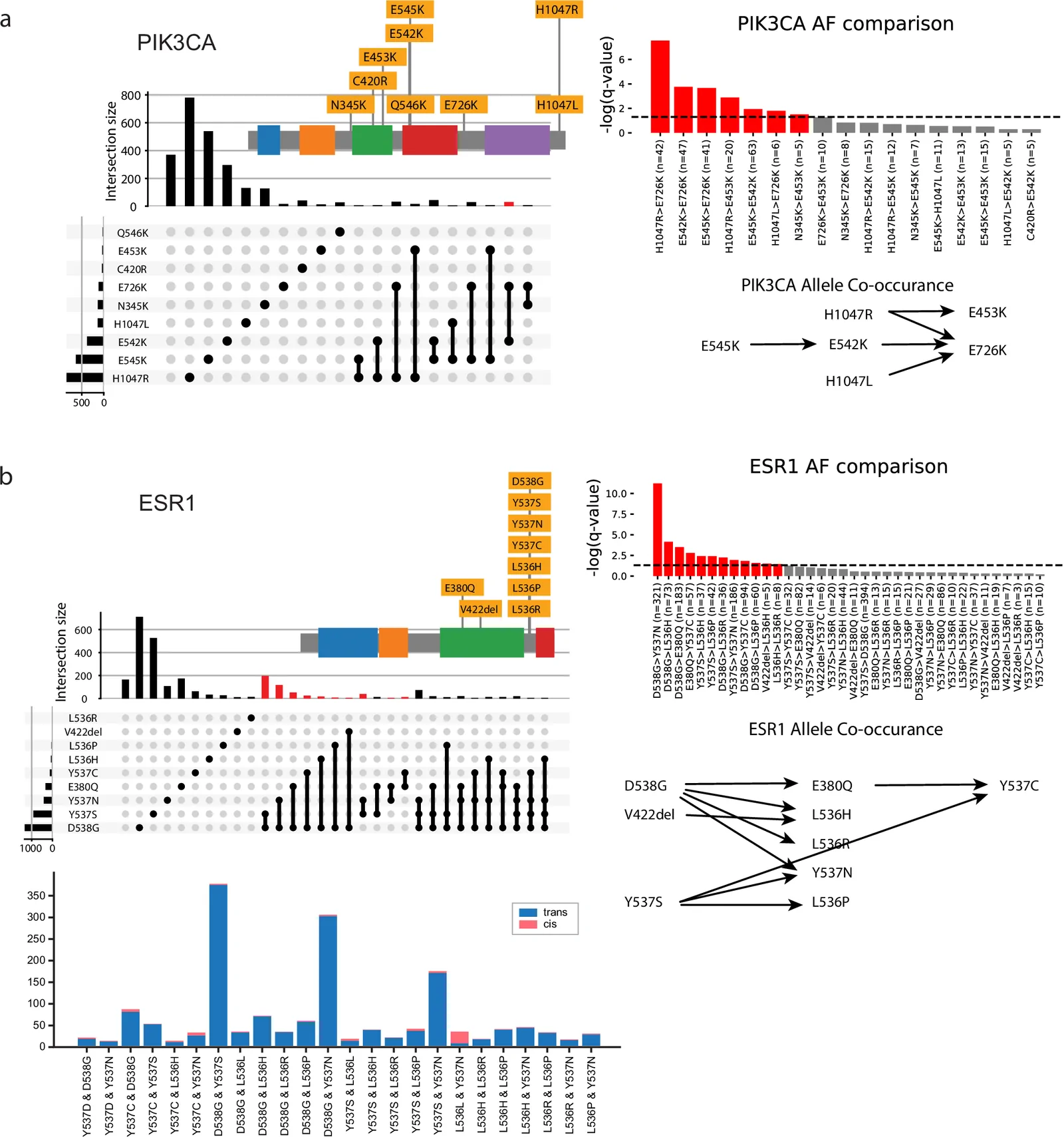
Co-occurrence of cis- and trans-mutations and interaction among common alleles. **a** Upset plot of co-occurrence of alleles in *PIK3CA* vs expected (left) and domination of allele fraction of specific alleles when occurring in the same sample (right). **b** Upset plot of co-occurrence of alleles in *ESR1* vs expected (left) and domination of allele fraction of specific alleles when occurring in the same sample (right). Bar chart for counts of read-based phasing of *ESR1* alleles, showing absence of cis-effects, with most alleles on different reads, except for DNP sites (bottom).

A large proportion of patients exhibited nonsynonymous alterations in *ESR1* (25%) and/or *PI3KCA* (34%). We evaluated if any of the commonly detected alleles preferentially co-occurred together in a single patient, which could suggest direct or indirect interaction between the protein changes (Fig. 3a, b).

Overall, ~94% of patients (3016 of 3218) with nonsynonymous *ESR1* mutations had one of the 9 most common *ESR1* alleles, suggesting their strong fitness advantage under therapy (Fig. 3b). Interestingly, ~40% of them (1217/3016) had more than one of the common alleles, which is consistent with resistance events occurring independently in multiple tumor sites in the body. We tested whether any of the mutation pairs co-occurred together more than expected by chance (Methods). Indeed, 10 mutation pairs significantly co-occurred (*q* < 0.05), with the most significant pair being D538G-Y537S, occurring in 14.4% (394 / 2729 in ER+) of ESR1 mutated patients vs. 9.5% (270/2729 in ER+) expected by chance (*q* = 0.003) (Fig. 3b). Interestingly, when we evaluated the available phasing information from the reads of cfDNA samples, nearly all proximal *ESR1* mutations showed trans- behavior (i.e., were present on different alleles), apart from a modest percentage of di-nucleotide substitutions (i.e., DNPs) appearing as adjacent single nucleotide cis-events (Fig. 3b). We therefore hypothesized that this enrichment of co-occurrence could be explained by a common focal amplification of the *ESR1* locus in the ER+ cases which would increase the amount of *ESR1* DNA and hence increase the chance of co-occurrence of the most common alleles. We did not have detailed amplification data to confirm this hypothesis, however, an average gain of 2.5x copies of the locus could theoretically explain all the significant co-occurrences due to single allele population frequencies. Such a level of *ESR1* amplifications is consistent with reported proportions in clinical samples^46,47^.

The most frequently detected *PIK3CA* mutations were H1047L, E454K, and E542K (Fig. 3a). Unlike *ESR1*, *PIK3CA* mutations have been described as clonal events, but also occur as acquired or emerging subclonal alterations in the setting of resistance^48^. We identified a pair of variants that significantly co-occurred (E542K and E726K), that could hint at cis-cooperation of these mutations, especially as the *PI3KCA* locus is seldomly amplified^49^. This is consistent with prior studies which have shown that patients with cis *PIK3CA* mutations exhibited decreased sensitivity to PI3K inhibitors^49^. Whether the same is true for double trans-*ESR1* mutations remains unknown. In *ERBB2*, the most frequent mutations were *V777L* and *S310F*. Interestingly, there were very few co-occurring mutations in this gene, suggesting that acquiring another mutation does not confer additional fitness advantage.

### Clonal co-occurrence of somatic drivers

**Fig. 4:**
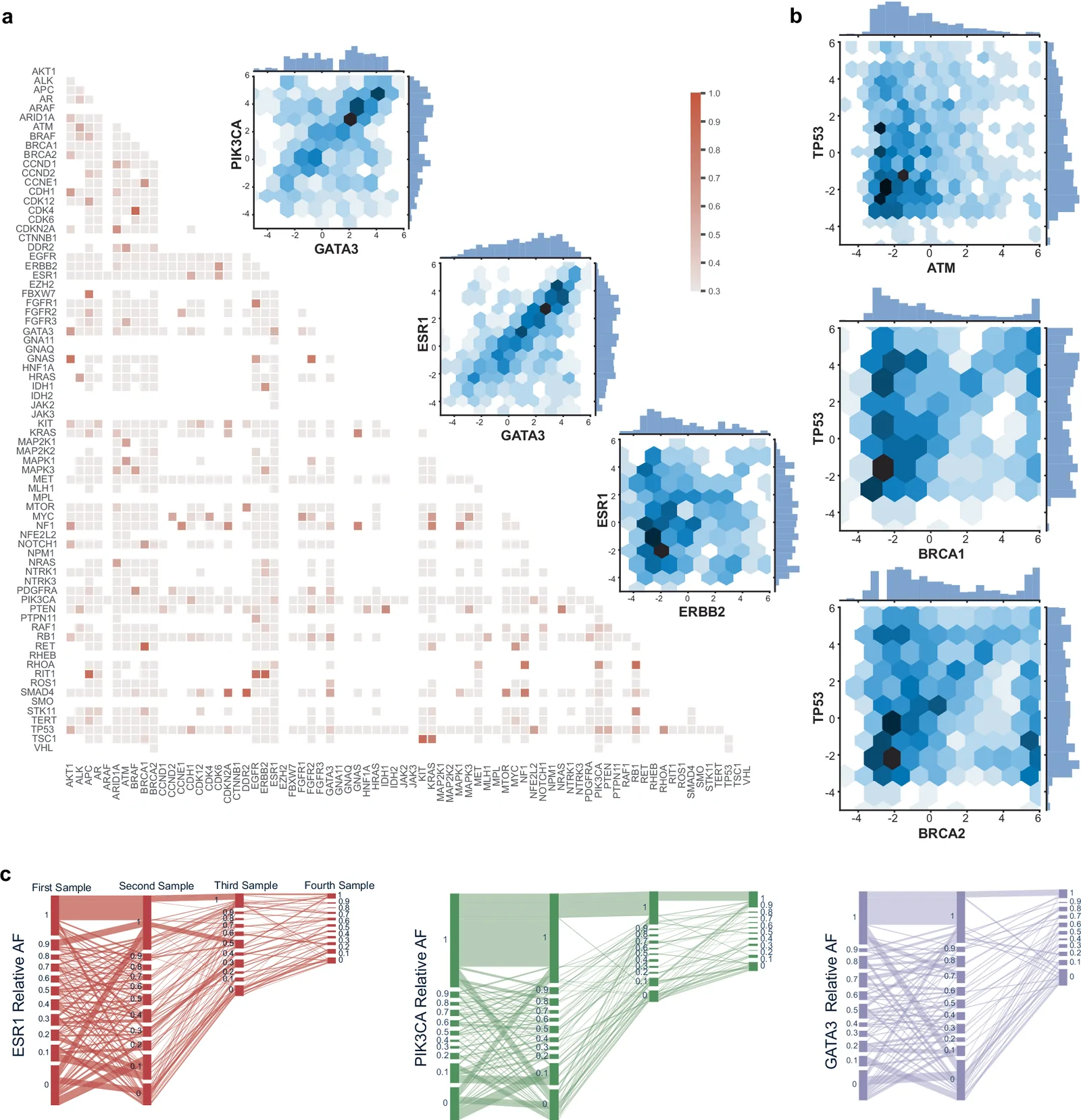
Clonal Frequency correlation and dynamics between timepoints. **a** Spearman’s correlation between log2(AF) of co-occurring mutations in the same samples with at least 15 co-occurrence samples. Insets showing the actual distribution of log2 allele fractions for PIK3CA-GATA3, ESR1-GATA3 and ESR1-ERBB2 gene pairs. **b** Distribution of co-tracking allele fractions in triple negative tumors, showing a high prevalence of putatively germline causal mutations. Insets showing the actual distribution of log2 allele fractions for TP53-ATM, TP53-BRCA1 and TP53- BRCA2 gene pairs. **c** Clonal dynamics between timepoints with the same patient showing an increase in clonal fraction of resistance mutations over time.

Typical clonal analysis in whole-exome and whole-genome sequencing data would entail reconstruction of absolute copy number, purity and cancer cell fractions (CCFs) of individual somatic mutations followed by identifying clones by clustering these events based on their CCFs^50,51^. Our panel data do not provide detailed genome-wide copy-number information, however the large cohort size allowed us to correlate the AF of somatic mutations in co-occurring driver genes. We evaluated the joint distributions of AFs for alterations across the 74 genes on the panel (Fig. 4a). After accounting for differences in cfDNA purity by dividing the mutation AFs by the highest AF in each sample, unexpectedly, 67 gene pairs had statistically significant correlations on both Spearman and Pearson tests (p < 0.001) and correlation values above 0.4, while the median correlation across all 982 pairs with at least 15 co-occurrences in the same sample was ~0.056. For example, *ESR1* mutations showed significant correlations with 6 genes (*CDH1, CDK6, GATA3, PIK3CA, RHOA*), the strongest correlation being with *GATA3* (Spearman rho = 0.546, *p* = 8.6×10^−39^). These findings are hypothesis generating, and suggest the mutations may be co-occurring in the same clones and thus potentially synergistic. We have evaluated other explanations of this high correlation behavior, such as biases by purity of the samples, but the majority of the existing gene pairs show very low correlation even subject to same sample purity constraints. Since these samples were obtained at metastatic and often treatment-resistant stages, this fitness advantage may represent cooperating resistance events. The potential interaction of *GATA3* and *ESR1* is interesting, given *GATA3* is a transcription factor that interacts with ER and FOXA1 on the same axis, and mutations in both *ESR1* and *GATA3* have been invoked as mechanisms of endocrine resistance and more aggressive phenotypes (e.g., *GATA3* mutations and lung metastases in endocrine-resistant HR+/HER2- breast cancer)^52,53^. *GATA3* mutations also showed a very high correlation with *PIK3CA* mutations, suggesting interaction of these genes in the setting of *ER*+ MBC. Also interestingly, in TCGA and other primary breast cancer analyses, *GATA3* and *PIK3CA* mutations have been described as mutually exclusive; thus this co-occurrence speaks to a distinct genomic landscape in advanced disease, which we have separately described in clinical analyses of patient cohorts resistant to endocrine therapy^54,55^. Other genes showed little correlation in AFs even with high mutual co-occurrence, suggesting mutual exclusivity of clones and resistance behavior (e.g., *EGFR* and *ESR1*, *ERBB2* and *ESR1*, etc.). Notably, mutations in the TNBC subtype showed a potential germline component in a number of predisposition genes (*BRCA1/2, ATM*) with AF distributions that appeared independent of somatic events in the same genes (Fig. 4b).

### Common mutations exhibit differential temporal clonal growth patterns

Our cohort had a substantial representation (1315, 11.5 %) of patients with detectable variants and multiple cfDNA samples collected over time, which allowed us to evaluate ctDNA load and clonal dynamics of the most prevalent driver genes and resistance mutations in MBC (Fig. 4c). We had compared the AFs of the most common mutation events across existing serial samples to the top AF of a somatic mutation observed consistently across patients’ samples, thus, a relative AF (rAF) = 1 would represent a clonal non-amplified event. In the ER+ cohort, almost a quarter of patients had an *ESR1* mutation as the top clonal variant (i.e., the highest AF among all the cfDNA mutations). In evaluating these mutations via longitudinal sampling, a substantial proportion of the *ESR1* mutations exhibited clonal expansion with increased relative AFs in the serial samples, suggesting selection for these events under treatment. PIK3CA and GATA3 mutations exhibited similar increase in rAFs over time, with emergence of novel resistant clones and expansion of earlier preexisting clones often reaching nearly clonal AFs in the 2^nd^, 3^rd^, or 4^th^ timepoint.

### Intergenomic clonal dynamics impact therapeutic response and survival

**Fig. 5:**
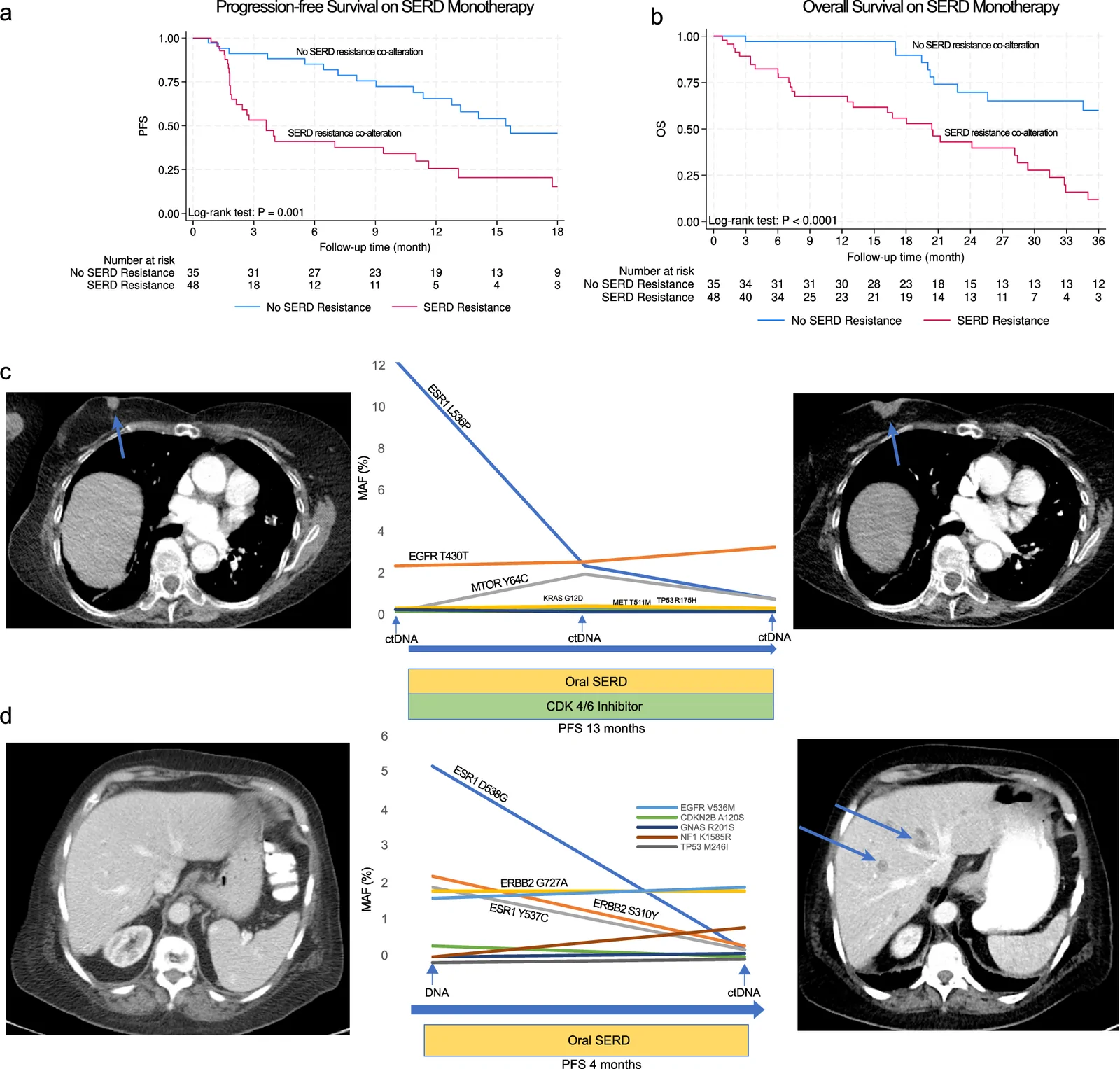
Coexisting resistance alterations, survival, and index patients. **a**, **b** Patients that received targeted SERD monotherapy were stratified based on the presence or absence of co-occurring alterations associated with SERD resistance. Median PFS 3.6 vs 15.4 months in patients with and without resistance co-alterations, respectively (HR 2.3; 95% CI 1.25–4.19; Cox Proportional Hazards *p* = 0.007). Median OS was 20.4 vs. 59.6 months in patients with and without resistance co-alterations, respectively (HR 2.7; 95% CI 1.02–7.24; Cox Proportional Hazards *p* < 0.001). **c**, **d** Two representative patients who had activating *ESR1* mutations and received an oral SERD on clinical trial. Patient 5c did not have a co-occurring resistance mutation and had a PFS of 13 months. Patient 5 d had co-occurring activating *ERBB2* mutations. While the VAF of the *ESR1* mutations decreased while on a SERD, the *ERBB2* G727A mutation VAF did not decrease accordingly, and the patient experienced progressive disease with new liver metastases on her next restaging scan 4 months later.

Amongst the analyzed cohort were a subset of patients with documented cfDNA testing that had been treated at the Massachusetts General Hospital (MGH). These patients were analyzed amongst additional patients treated at the MGH with documented cfDNA testing, and among whom 83 patients had received selective estrogen receptor degrader (SERD) monotherapy, either fulvestrant (*n* = 35) or one of several oral SERDs on clinical trial (*n* = 48). Given there are multiple ongoing clinical trials evaluating oral SERD monotherapy, one of which recently led to approval of elacestrant in ER+/HER2- *ESR1*^mut^ MBC, we sought to evaluate the association of co-variants on the efficacy of SERD monotherapy, and we hypothesized that variants associated with poorer SERD response would be associated to poorer progression-free survival (PFS) and overall survival (OS), in the absence of combination therapy. Of note, most patients were treated prior to CDK4/6 inhibitors becoming standard-of-care as part of first-line ER+ MBC therapy. Among these 83 patients, 48 had cfDNA-detected alterations associated with poorer SERD response, based on published in vitro and clinical evidence (Table 2); these variants were associated with significantly shorter median PFS and OS compared to patients who did not have coexisting SERD resistance-associated mutations (Fig. 5a–d, Suppl. Fig. 4–5 and Suppl. Data 5, 6). Median PFS was 3.6 vs 15.4 months in patients with and without resistance co-alterations, respectively (HR 2.3; 95% CI 1.25–4.19; *p* = 0.007). Median OS was 20.4 vs. 59.6 months in patients with and without resistance co-alterations, respectively (HR 2.7; 95% CI 1.02–7.24; *p* <0.001). Given the first oral SERD approval was for cancers with detectable *ESR1* mutations, we performed a separate analysis only for this patient subpopulation^29^. Amongst 34 patients with *ESR1* mutations, the 23 patients with co-occurring variants associated with SERD resistance had a statistically significantly worse median PFS [3.6 vs 13.2 months (HR 3.6, 95% CI 1.02–7.23, *p* = 0.045)], and worse median OS [20.5 months vs median not reached (HR 3.6, 95% CI 0.99–13.0, *p* = 0.050)]. Multivariate analysis showed no underlying effects from age at primary diagnosis, number of prior therapies, presence of visceral metastases, oral vs IM SERD, and prior CDK4/6 inhibitor use across these study groups. Two contrasting index patients with *ESR1* mutations who received an oral SERD (on clinical trial) are highlighted in Fig. 5c, d. Patient A had no alterations associated with poorer response, received combination therapy with a CDK4/6 inhibitor, and had a PFS of over 1 year, while patient B had a PFS of under 4 months. While they both had disease with ESCAT I *ESR1* mutations that decreased in response to targeted oral SERD therapy, patient B had additional resistance mutations in the form of activating *ERBB2* alterations, S310Y & G727A, the latter of which showed a VAF increase during treatment with SERD, close interval disease progression and poor survival. These findings suggest continued growth of resistance subclones, utilizing a pathway independent of ER signaling and thus less responsiveness to SERD therapy alone. The efficacy of targeted therapy, particularly monotherapy, may be limited in the setting of co-existing alterations that may confer resistance.

## Discussion

Our study characterizes the complex landscape of genomic alterations in advanced, heavily treated breast cancer and supports the use of targeted cfDNA sequencing as a practical noninvasive means of characterizing genomic evolution by capturing clinically significant alterations and accounting for spatial and temporal heterogeneity by, as previously shown, aggregating DNA shed from multiple lesions within the body. Our data shows the importance of serial sampling to evaluate emergent actionable alterations. The full cfDNA dataset is being released with this publication, which offers an invaluable resource for future genomic studies and planning of clinical trials in regard to patient candidacy and accrual, and for analyzing targetable alterations and co-occurring resistance variants.

Our dataset is the largest to date MBC profiling of cfDNA with 15,564 blood biopsy samples from 12,827 patients, nearly an order of magnitude larger than earlier studies. We have developed a cfDNA-based classifier to predict breast cancer subtypes from unannotated datasets, which may be used in further analyses with this and similar datasets. As a tool for clinical investigators, this resource represents patients with advanced disease from across multiple centers in the US and allows serial evaluation of the dynamics of actionable and resistance genes within and across breast cancer subtypes^56^.

Our data substantially increases the available genomic information for patients with MBC, including the recent releases of the MSK-MET cohort, consisting of over 25,000 solid tumor patient samples with metastatic disease (2609 of which are breast cancer) and the Hartwig Medical Foundation cohort of 5094 solid tumors (including 855 breast cancer)^57^. This data also significantly complements a recently published large tissue and cfDNA collection of MBC ( > 29,000 tissue biopsies and > 3000 cfDNA samples), bringing the total number of available cfDNA samples to almost 19,000^28^. A previously published tissue-based analysis of 1918 breast cancer samples demonstrated that genomic alterations were enriched in 32 genes in the metastatic setting, with spatially distinct and mutually exclusive patterns of resistance^25^. These findings show multiple spatially distinct mechanisms of resistance to endocrine therapy and highlight the importance of plasma-based genotyping to complement these tissue-based findings and capture this complex landscape in the advanced setting.

There are currently numerous genomically-targeted clinical trials in breast cancer, several of which have already led to new standards-of-care, as well as a wave of follow-up targeted therapy trials. In ER+/HER2- MBC, alpelisib and inavolisib are approved for use in the setting of *PIK3CA* mutation^58,59^, the oral SERD elacestrant with *ESR1* mutations, and most recently capivasertib if variants in *PIK3CA, AKT1, or PTEN*. While many *PIK3CA* mutations are truncal, *ESR1* mutations are almost always acquired under therapeutic pressure^18,19,29,60^. In order to detect targetable alterations, patients will likely require serial tumor genomic testing, most feasibly through cfDNA. Furthermore, multiple studies are evaluating drugs targeting individual mutations in *PIK3CA*, as well as further down the PI3K-AKT pathway (e.g., AKT inhibitors). In total, we anticipate a significant further increase in cfDNA analyses in advanced, pre-treated breast cancer, and this database will prove an important resource in contextualizing patient findings.

This database will also prove an invaluable tool to investigators planning genomically-targeted clinical trials, since in order to assess and predict accrual, it is important to know expected frequencies of targetable variants as well as alterations that may impede drug efficacy. As tissue biopsy is often unavailable in the advanced disease setting, this database would allow investigators to predict frequency and detectability of targetable alterations via cfDNA, informing future cfDNA-based clinical trials by estimation of eligible patient populations, based on genomic inclusion criteria, and resistance mechanisms that may impede efficacy.

Compared to tissue-based genomic profiling of primary breast cancer, we found significantly more alterations in clinically actionable genes in these advanced cancer samples, the majority of which were in the ER+ subtype. Given current drug approvals, ER+ patients are more likely to receive targeted therapies for prolonged periods of time, including SERDs, aromatase inhibitors, CDK4/6 inhibitors, PI3K inhibitors, AKT inhibitors, and PARP inhibitors. We hypothesize that prolonged time of targeted therapy may facilitate the development of multisite resistance as the cancer evolves.

We identified several groups of genes with highly correlated allele frequencies suggesting interaction of these alleles in the metastatic or treatment-refractory setting. The high abundance of suspected *cis*- vs. *trans*-mutations of *PIK3CA* highlight a potential synergy of the cis-mutations, while mutations in *ESR1* and *ERBB2* did not show strong evidence of allele *cis*-interaction, with almost no mutations in *cis*. Co-occurring mutations also appeared unique to the metastatic landscape, including *PIK3CA-GATA3* and *ESR1-GATA3*. These findings could inform future studies such as metabolomic analyses to understand mechanisms of these co-occurrence patterns.

In the EMERALD trial, which led to approval of elacestrant for ER+/HER2- *ESR1*^mut^ MBC, both the control (fulvestrant) and elacestrant groups experienced significant decrease in survival at start of therapy, which suggests baseline resistance in subpopulations in both groups. Given our observations in cfDNA, it is possible that inherent SERD resistance is due to activated resistance pathways outside the ER axis and SERD therapy may carry greater benefit when implemented prior to acquisition of alternative resistance mechanisms. Importantly, though we cannot infer immune pressure through cfDNA studies alone, we may use these findings to inform our future clinical studies using solid tumor profiling. The efficacy of oral SERDs with targeted combination therapy is currently being evaluated in multiple clinical trials^61–63^. Meanwhile, multiple studies are ongoing evaluating other approaches to targeted therapies, including PlasmaMATCH, which has reported clinically relevant activity in cfDNA-informed targeted therapy against *ERBB2* and *AKT1* mutations^15^.

As more agents are approved in MBC, the question of sequencing-informed therapy also becomes important. Identification of existing or emerging resistance mutations in cfDNA could be utilized as the basis for switching therapy early, before clinical progression^64–66^. The PADA-1 trial was the first of its kind to evaluate the clinical significance of changing therapies in response to resistance alterations in cfDNA. Patients on an aromatase inhibitor and the CDK4/6 inhibitor palbociclib with *ESR1* resistance mutations identified on cfDNA were randomized to continue therapy or change to fulvestrant with palbociclib. Median PFS was significantly improved in the patients who changed the endocrine therapy even when accounting for subsequent therapy modifications in the control arm^67^.

There are important limitations to consider in our study. First, our classifier was trained on genomic data. Thus, identification of subtypes could be skewed, relying more on commonly mutated genes in the corresponding subtype. This study also used targeted sequencing on predominantly known cancer genes, which allowed for deep cfDNA profiling, but could lead to missed novel resistance drivers and actionable alterations. Furthermore, this cfDNA platform does not include the analysis of a germline control, which could potentially result in rare germline mutations being reported as somatic, and does not use peripheral blood mononuclear cells to detect CH, and thus there is a possibility of CH mutations being called as tumor-associated alterations^68^. This data also does not provide deep clinical annotation, which limits interpretation of results in the context of specific type and line of therapy and clinical extent of disease. Guidelines have also been further developed since the start of this study, thus changing the indications for individual testing. Finally, ESCAT I classification is assigned to therapies that have been approved as genomic-targeted therapies. The majority of targeted therapies in MBC are approved for ER+/HER2- disease, thus this subtype had the largest percentage of ESCAT I/II variants. In order to increase the number of ESCAT I/II variants in HER2+ and TNBC MBC, targeted therapies must be developed and studied, using this dataset as a tool for further development.

In summary, this study significantly expands upon previously established data by describing the genomic landscape of cfDNA in advanced disease on a uniquely large cohort of over 12,500 patients. We describe significantly increased abundance of actionable mutations and likely resistance mechanisms in advanced disease with future clinical implications for targeted therapy treatment guided by cfDNA diagnostics. Our study provides a large repository of advanced breast cancer genomic profiling that is publicly available for the research community and to inform clinical trials.

## Methods

### Sample collection and genomic analysis

This study complies with all relevant ethical regulations. Peripheral blood samples from patients with advanced cancer were collected in Streck tubes and submitted for targeted cell-free DNA sequencing using a 73–74 gene next generation sequencing (NGS) panel (Guardant360, Guardant Health, Inc; Redwood City, CA, USA). This CLIA-certified, CAP-accredited, NYSDOH-approved clinical assay detects somatic single nucleotide variants (SNVs) in 73–74 genes (panel composition changed during the course of the study), and indels, somatic copy-number gains, and fusions in a subset of these genes as previously described. The data was provided as a table of point mutations, structural variants, and copy number amplifications in a mutation annotation file type format (Suppl. Data 2). At allele fractions > 0.25% for mutations and fusions, > 0.20% for indels, and copy numbers > 2.24 copies, the analytical sensitivity of this assay is ≧ 95% and specificity is > 99.99%^24^. A de-identified genomic database of these clinical results was queried for genomic alterations identified in samples with a diagnosis of breast cancer analyzed between November 2016 and May 2020 in accordance with an IRB-approved protocol waiving the need for informed consent for de-identified data analysis.

All samples and results met standard requirements including quality control metrics for clinical testing for CLIA-approved tests, and the methodology for the testing itself, including technical details of analytical process and validity, have been previously published^60^.

### Comparison of Guardant assay results to TCGA and METABRIC

In order to determine the genomic differences between primary and advanced breast cancer, we compared the expected prevalence of mutations in our subtype cohorts to two genomic databases of primary breast cancers: The Cancer Genome Atlas (TCGA) and the Molecular Taxonomy of Breast Cancer International Consortium (METABRIC). TCGA is a collection of genomic data obtained from whole exome sequencing of over 800 primary breast cancers, whereas METABRIC is a data repository of genomic information obtained via targeted sequencing from almost 2000 primary breast cancer samples. We defined statistically significant differences as a false discovery rate (q) of ≤ 0.05.

### cfDNA-based classifier for predicting breast cancer subtype

A hierarchical tree (2 layers, Fig. 1d) of 3 Random Forest Classifier (RFC) composites, each composed of 10 RFCs, was used to classify the ER and HER2 statuses of unannotated patients. The first RFC composite was trained on 392 patients with existing clinical ER annotations. The patients were split into 10 disjoint groups each used to train one of the 10 RFCs in the composite. The composite ran patients through each of the 10 RFCs and averaged their outputs. This composite strategy was done to minimize overfitting. The input matrix included 74 genic point mutation features, 73 copy number amplification features, and 19 drug treatment features (total 166). The drug treatment features included annotated drug names (Ibrance/palbociclib, Faslodex/fulvestrant, Kisqali/ribociclib, tamoxifen, etc.). The data was represented by Boolean values denoting whether a point mutation was detected at the feature gene, a copy number amplification was detected at the feature locus, or the treatment annotations included the drug feature. For 312 patients with missing treatment annotations, we set the drug treatment features to 0.5. Notably, ERBB2 amplification and anti-HER2 drugs (trastuzumab, pertuzumab, lapatinib, etc.) were excluded from the ER features since they are highly indicative of HER2 status, which was the domain of the second layer. Once patients were classified as either ER+ or ER-, they were again classified for HER2 status using similar classifiers specifically trained on either ER+ or ER- patients. For these classifiers, the ERBB2 amplification and anti-HER2 drugs were added back to the trained features.

### Co-occurrence of cis- and trans-mutations and interaction among common alleles

Permutations of assignment labels were used to identify enriched co-occurrences of point mutations in common driver genes. Point mutations were assigned independently to random patients according to their marginal frequencies, and co-occurrence rates in the simulations were compared with observed values. *P*-values for each pair of point mutations were calculated as number of simulations with equal or higher rate of co-occurrence than observed plus one divided by the total number of simulations plus one. Corrected q-values were calculated using Benjamini-Hochberg multiple hypothesis test correction. Cis and trans mutations in close proximity were called by phasing co-occurrence on individual reads. To identify enriched allele fraction domination within pairs of co-occurring point mutations, pairs of point mutations were tested against the null hypothesis that either mutation is equally likely to have an allele fraction greater than the other. Pairs of point mutations were sorted according to their observed allele fractions, then *p*-values were calculated as the sum of the tail greater or equal than the observed domination count of the symmetric binomial distribution (i.e., binomial probability = 0.5). Again, corrected q-values were calculated using Benjamini-Hochberg multiple hypothesis correction.

### Clonal co-occurrence of somatic drivers

Joint distributions of AFs for all co-occurring alterations across the 74 genes on the panel were constructed, and both Pearson and Spearman’s correlation between log_2_(AF) of co-occurring mutations in the same samples with at least 15 co-occurrence samples were measured. Statistically significant correlations after Benjamini-Hochberg multiple hypothesis test correction were identified. The median correlation across all pairs with at least 15 co-occurrences in a sample was 0.056. We evaluated other explanations of high correlation behavior, such as biases by purity of the samples, but the majority of the existing gene pairs show very low correlation even subject to same sample purity constraints. The distribution of pairwise Spearman’s correlations of AFs, after correcting for the cfDNA purity by dividing by highest AF in the sample, was plotted as all gene-by-gene heatmap with the density representing the correlation value for co-occurring gene pairs. Distribution of co-tracking allele fractions in triple negative tumors showed a high prevalence of putatively germline causal mutations.

### Patient selection and statistical analysis of survival dynamics on targeted therapies

Within the analyzed cohort of 12,827 patients, there was a subset of 459 patients who had been treated at the Massachusetts General Hospital, and who had consented to have their medical information used for research purposes. Medical records were de-identified prior to data analysis. Patients who had received SERD monotherapy were selected for survival analysis. Mutations conferring SERD resistance were identified via cfDNA collection. PFS and OS were calculated via the Kaplan-Meier method, and survival curves between patients with and without co-existing resistance mutations were compared using logrank testing. Day 1 was the start date of SERD monotherapy. The association of risk factors and outcomes were estimated using the Cox proportional hazards method. All analyses were performed using STATA, version 16 (StataCorp LLC).

### Statistics and reproducibility

No statistical method was used to predetermine sample size. No data were excluded from the analysis. The samples were not randomized. Statistical analysis was performed using python packages scipy-1.16 and statsmodels-0.14.

### Reporting summary

Further information on research design is available in the Nature Portfolio Reporting Summary linked to this article.

## Supplementary information

Supplementary Information

Description of Additional Supplementary Files

Supplementary Data 1

Supplementary Data 2

Supplementary Data 3

Supplementary Data 4

Supplementary Data 5

Supplementary Data 6

Reporting Summary

Transparent Peer Review file

## Source data

Source Data

## Data Availability

The full Guardant360 dataset used in the manuscript is available in the Supplementary Data. Source data are provided with this paper.

## Code availability

The code used to analyze the cfDNA data is available on GitHub at https://github.com/getzlab/BRCA_ctDNA_Landscape.
