# Supplementary material for: Actionable genetic landscape and dynamics in circulating DNA from 12,827 patients with metastatic breast cancer
Source: Nat Commun. 2026 Sep 22;17:11023. doi: 10.1038/s41467-026-75976-9 (PMC13687546; doi:10.1038/s41467-026-75976-9)
Supplement: Supplementary file 1 [file 41467_2026_75976_MOESM10_ESM.pdf]

nature portfolio

Peer Review File

Actionable genetic landscape and dynamics in circulating DNA from 12,827 patients with metastatic breast cancer

Corresponding Author: Professor Gad Getz

This file contains all reviewer reports in order by version, followed by all author rebuttals in order by version.

Version 0:

Reviewer comments:

Reviewer #1

(Remarks to the Author)
Leshchine, Medford, and Cha et al. present a large-scale analysis of cell-free DNA (cfDNA) in 12,827 metastatic breast cancer patients. It's a large cohort analysis that revealed the genetic alteration landscape by identifying Guardant360 assay. The manuscript is well written and can serve as an important repertoire for further development, especially given the extent of the data and the number of samples collected. While the manuscript describes the results meaningfully, I have some major concerns:

Major concerns –
1. The authors should provide a detailed schematic of the samples. Current overview figure given in Figure 1 is not sufficient. It does not describe (in figure or text) the exact time intervals used for collecting the samples. For example, what are the major clinical milestones - which ones are collected before initiating the new therapy, what was the time scale of the disease progression, or the resistance monitoring. Added clarity will help the readers to understand the scale of the experiment.
2. The demographic information, and age might add another dimension in understanding the factor towards the analysis.
3. The prediction scores using random forest classifiers are described without specified uncertainty scores. Specifically, TNBC scores are 73% which seems on the lower side. It would be great to judge the performance in comparison with any external database, albeit with a lower number of samples.
4. The tumor suppressor mutations and oncogenes heatmap (Figure 1d) does not explain the color schemes in the legend. Adding to this maybe stratification of the type of mutation (passenger vs driver) could be interesting to know. Overall the readability of Figure 1d should be improved.
5. Figure 1e describes the flow of the algorithm. But it would be better to use some notations to denote the variables, the algorithm is predicting and the input feature being used. Then the rest of the discussion about prediction probability becomes clearer.
6. In the similar direction Figure 1f-h is very hard to comprehend. We understand that allele frequency is related to mutation rate, and there are mutation events related to particular genes. The probability of detection is hardly meaningful and looks like a dark bar. The authors should provide a breakdown of why specific mutations drive classification decisions. Use of log-scale might help with low-frequency mutations. Additionally, any external classification (for example detection by biopsy), would be good to know as a baseline of the prediction. Adding some overall numbers or figures (such as violin plots) would be helpful to understand the distribution of the probabilities across 3 different types of cancers.
7. Figure 2a is very informative in knowing the actionable cancer types. Is it possible to add the longitudinal axis to this. Currently we don't see the time to be a factor of change in the bar plots. Additionally, the relation to the frequency of mutation is not clear to me. For example, are low frequency mutations (rare) less actionable? I am also a little concerned about the class imbalance in the study, for example ER+ patients overwhelm the whole study. Is it possible to sample the dataset to make a balance representation and then evaluate the effects?
8. Cis vs trans mutations are very important phenomena to study and can have practical impact on tumor-growth and treatment strategy. While figure 3 describes the major observations, I feel the study itself is not well-explored. For example, it is hypothesized that trans-ESR1 mutations have worse therapy outcomes due to independent resistant clones, but no survival analysis is done directly. Moreover, having a parallel copy number analysis can validate this directly at least on some selected patients. Another line of interesting validation could be functional – one a model organism the authors can design CRISPR-engineered breast cancer cell lines harboring single vs. multiple PIK3CA mutations as proxy in same vs different alleles and directly measure the activation of PI3K inhibitors. A differential analysis would directly prove if their

hypothesis is valid. Another potential analysis could be checking the transcriptional landscape of cis vs trans mutation.
9. Clonal occurrence between 67 pairs of mutations is an interesting observation. PIK3CA-GATA3 co-occurrence pattern is unique to MBC samples. Is it possible to validate if this is due to therapy-driven selection. Following my last suggestion, is it possible to do multi-region tumor sequencing from metastatic lesions to determine if the co-mutations occur in distinct clones? This analysis can also be used to actually model co-evolution. Can this be modeled as a joint distribution of mutation where marginal distributions are fixed (e.g. they are Poisson samples etc.)

Expanding on PIK3CA-GATA3 mutation, that co-occurs significantly according to MBC patients – does it also reshape the metabolomics landscape - e.g. shift tumors toward glycolysis? Performing metabolomic analysis could reveal this.

10. While SERD therapy response is revealing, it relies solely on cell-free DMA to infer the response. It is possible to identify some key samples and do sc-DNA-seq of serial biopsies – this helps in differentiating between pre-existing clones and clones generated due to therapy.

11. The survival analysis does not take into account the tumor microenvironment influences at all. While it is not directly part of the study, but, could significantly change the clonal selection.

Minor concerns –
- Typo line 392 "increase increase"
- Some sentence are not clearly written – e.g. "as well as further down the PI3K-AKT pathway as AKT inhibitors." – what does it mean? or "which may suggest the impact that time on treatmen..t".

Overall, I feel this paper will provide useful resources to the community, however it lacks functional validation to back the claims. Complementarily the manuscript also does not make a principled mechanistic model of mutually dependent clonal evolution. The breadth of the analysis is appealing but it lacks depth (in both method development or building computational models). To make the present manuscript impactful, I strongly believe, the authors should expand on either or both of these directions.

(Remarks on code availability)

Reviewer #2

(Remarks to the Author)
Leshchiner et al presents the largest dataset to date of cfDNA samples from patients with metastatic breast cancer (and possibly with any cancer type), comprising 15,564 samples from 12,827 patients analyzed using the Guardant360 assay. The authors report comprehensive genomic profiling of actionable alterations across breast cancer subtypes, develop a novel cfDNA-based classifier to predict breast cancer subtypes, analyze resistance mechanisms, examine co-occurring mutations, and study clonal dynamics through longitudinal sampling. The work provides valuable insights into the genomic landscape of MBC and has potential implications for clinical trial design and personalized treatment approaches. However, there are several concerns about the manuscript:
Major Concerns
1. The study does not validate whether the detected cfDNA mutations (e.g., ESR1, PIK3CA) are somatic or reflect tumor biology. Germline variants, clonal hematopoiesis (CHIP), or mutations from normal/premalignant tissues (e.g., TP53 in aging) may confound the results. It would be helpful to perform orthogonal validation using matched tumor tissue biopsies to assess concordance between cfDNA and tumor mutations, particularly for targetable variants (e.g., ESR1 ligand-binding domain mutations).
Or if there are any report on the validation Guardant 360 assay (even on other tumor types), the authors can also use the data to give an estimation that what percentage of cfDNA mutations are somatic.

2. The assay cannot distinguish germline mutations (e.g., BRCA1/2) or CHIP-derived variants (e.g., TP53, ATM). This is critical for interpreting targetability (e.g., PARP inhibitors require germline/somatic BRCA status). Among these sources of non-somatic mutations, those from CHIP can be filtered by sequencing the WBC DNA. However, the authors didn't perform this analysis, either. I suggest to discuss limitations of relying solely on VAF thresholds (e.g., 40% for BRCA1/2 germline/somatic distinction) without paired germline testing. CHIP-filtering algorithms (e.g., using variant allele frequency [VAF] and gene-specific CHIP patterns) could also be applied for this issue.
3. The authors should validate the classifier in an independent cohort with pathology-confirmed subtypes. In the current manuscript, the performance of the classifier is based on cross validation, which lead to concerns of overfit. It would be better to use half of cases to train the classifier and use the rest for an independent validation. A weaker validation (if the cases available for this analysis is too limited) is to apply the classifier to the rest cases without label of subtype. And the clinical features of TNBC cases identified by the classifier should be compared to those from TNBC cases from independent cohorts and confirmed by pathology.
4. I suggest to include more details like cfDNA yield, tumor fraction thresholds, or library preparation QC metrics. Clarify inclusion criteria (e.g., minimum cfDNA concentration, tumor fraction thresholds) and their impact on variant detection.

5. Statistical Thresholds: Using FDR $q < 0.1$ is overly permissive. Adopt $q < 0.05$ or justify the lenient threshold with sensitivity analyses.

6. The ESR1 Mutations and ER+ Classification seems circular reasoning: ESR1 mutations are both a classifier feature and a claimed biomarker of ER+ status. Try retraining the classifier excluding ESR1 mutations to evaluate its dependency on this feature.

7. The gene pair correlation results are interesting (Fig. 4a). However, the authors should acknowledge the hypothesis-generating nature of these observations and avoid causal language like "synergistic" interactions (e.g., GATA3-ESR1)
8. The authors admitted that copy number calls (e.g., FGFR1/CCND1 amplifications) are unreliable in low tumor fraction samples. They should restrict analyses to high tumor fraction samples or validate Guardant360 CNV calls against orthogonal methods (e.g., FISH) or use Guardant data (if any) to show the validation.

5. The MGH Cohort is too small (n=48). Survival differences (PFS/OS) in SERD-treated patients are underpowered and lack multivariate adjustment (e.g., prior therapies, CDK4/6i use). The authors should try to expand the cohort or contextualize findings as exploratory. A big advantage of this manuscript is the huge size of the cohort. However, if the major/clinically important conclusions are from very small fraction of cases, the power of the huge cohort could be compromised.

Minor concerns
1. Figure 1d (Genomic Landscape): Overly dense and lacks clarity. Maybe it's better to split into subpanels (e.g., mutation types, CNVs) and define "TS/Onc" categories.

Figure 3 (Co-occurrence Analysis): Poor resolution in Upset plots; cis/trans phasing lacks statistical rigor.

2. Methods Description

Classifier Training:
Feature selection for the Random Forest classifier (e.g., treatment annotations) is unclear.

Clonal Analysis:
Methods for purity correction (e.g., using "highest AF" as a proxy) and clonal/subclonal categorization are vague.
3. It's better to specify data types (raw FASTQ, processed mutation lists).

(Remarks on code availability)

Reviewer #3

(Remarks to the Author)
The report by Leshchiner et al. characterizes the mutational spectrum of a large cohort of tumors in patients with metastatic breast cancer tested by Guardant using the Guardant360 mutation panel. The sheer size of this cohort is impressive and enables a very comprehensive cataloguing of the most common and most highly coexpressed mutations from amongst those on the panel.
The strengths and novel findings of the study include:
1. The use of a relevant panel of 74 clinicallysignificant genes
2. Depth of sequencing enabling precision
3. Comparison to mutational spectrum in primary tumors from METABRIC and TCGA
4. Careful work looking at co-occurrence of cis and trans mutations, though I am concerned that the "interaction" is not a true interaction but rather correlation - - need to clarify that statistically.
5. Significant correlations between ESR-1 mutations and 6 other genes - - CDH1, CDK6, GATA3, PIK3CA and RHOA. Though the clinical and biological significance of this phenomenon has yet to be elucidated

Despite these strengths, there are numerous issues that limit the generalizeability and utility of the data that is presented.

1. The ctDNA platform does not subtract germline mutations or CHIP. The latter may be particularly problematic in causing overestimation of the mutational burden.
2. The data is, for the most part, descriptive, and largely confirms prior findings in albeit smaller datasets.
3. The clinical characteristics of patients in the cohort is not described, nor does it seem available for the analysis. Presumably, this is a cohort of patients selected by their clinicians to have the Guardant360 test performed. Given the timeframe in which the cohort data was collected, starting in 2017, this was a very early timepoint during which guidelines did not recommend mutation testing, so a very select group would have had this done. The possible bias is seen in the extremely high frequency of samples harboring at least one mutation Thus there is inherent selection bias – the extent to which this skews the data from what would be seen in an unselected "all comers" type of study is not clear.
4. Moreover the cohort lacks clinical annotation, and thus we are not able to interpret the development of mutations in the context of clinical extent of disease, type of therapy (both over the course of disease and immediately prior to the test) or line of therapy, all of which would be helpful in understanding what the mutational spectrum is telling us about both intrinsic and acquired resistance.
5. The small subcohort with clinical annotation appear to be all derived from a clinical trial of elacestrant, which is an even more highly selected group of patients. This data, while interesting, does not seem appropriately included in this manuscript and would be better placed in a separate report as its relevance to the questions being answered in the larger cohort is unclear.

6. There are numerous portions of the manuscript that describe potential uses for ctDNA in metastatic breast cancer, recommendations from guidelines committees including ASCO, and other editorial type comments that do not seem to be relevant to the data being presented.
7. It is not possible to directly measure tumor evolution under pressure of treatment since treatment information is not available. There are sentences (eg 308-309) suggesting this without having any data to back it up.
8. Its difficult to understand the clinical value of the ER/HER2 predictor. The vast majority of patients, at diagnosis of metastatic disease, have a tumor biopsy in which receptors are measured. This is used for treatment purposes. The predictor developed by the authors has only 75% accuracy, which is insufficient to replace a biopsy. Moreover, when tumors become resistant to therapies that are targeting these receptors, the receptor expression doesn't change, just the function changes. Thus, a predictor of sensitivity to ER or HER2-directed therapy would be useful; simply predicting the subtype is unlikely to have particular utility in improving outcome.

(Remarks on code availability)

Version 1:

Reviewer comments:

Reviewer #1

(Remarks to the Author)
The authors have satisfactorily responded to the comments during the first round of review, improved the manuscript and better positioned it as a resource paper.

(Remarks on code availability)

Reviewer #2

(Remarks to the Author)
The authors have made substantial improvements, but some critical issues remain:
1.Germline vs. Somatic / CHIP Distinction: The authors mentioned that 18% of plasma mutations cannot be identified in WBC sequencing. Paired plasma-tissue discordance exists due to heterogenenity. These partially explained the issue, but cannot solve the problem. This remains the most critical weakness. We still don't know, what proportion of TP53 mutations might be CH-derived? Etc. This needs additional analysis or at minimum, much stronger language in the Discussion about how CH/germline contamination may inflate certain mutation frequencies and limit clinical interpretation.
I would suggest: (1) Add Supplementary Figure showing VAF distributions for commonly mutated genes; (2)Perform sensitivity analysis excluding CH-prone genes (TP53, ATM, BRCA1/2, CHEK2); (3)Re-calculate actionability rates with/without these genes; (4)Add prominent Discussion paragraph acknowledging how CH may inflate certain mutation frequencies

2.Clinical Context/Selection Bias:
a)Add to Discussion: Clear statement that cohort represents selected patients (high tumor burden, high cfDNA yield, which could include more progressive disease)
b)Compare cohort characteristics to published MBC populations
c)Acknowledge generalizability limitations more prominently
3.Language:
a)Change "interaction" to "correlation/co-occurrence"
b)Add "hypothesis-generating" qualifications to correlation findings

(Remarks on code availability)

Reviewer #3

(Remarks to the Author)

(Remarks on code availability)

Version 2:

Reviewer comments:

Reviewer #2

(Remarks to the Author)
The authors have thoroughly addressed all of my previous concerns.I recommend acceptance of this manuscript.

(Remarks on code availability)

Open Access This Peer Review File is licensed under a Creative Commons Attribution 4.0 International License, which permits use, sharing, adaptation, distribution and reproduction in any medium or format, as long as you give appropriate credit to the original author(s) and the source, provide a link to the Creative Commons license, and indicate if changes were made.
In cases where reviewers are anonymous, credit should be given to 'Anonymous Referee' and the source.
The images or other third party material in this Peer Review File are included in the article's Creative Commons license, unless indicated otherwise in a credit line to the material. If material is not included in the article's Creative Commons license and your intended use is not permitted by statutory regulation or exceeds the permitted use, you will need to obtain permission directly from the copyright holder.
To view a copy of this license, visit https://creativecommons.org/licenses/by/4.0/

Point by point Response

We thank the reviewers for a detailed and constructive critique of our manuscript.

Reviewer #1 (Remarks to the Author):

Leshchiner, Medford, and Cha et al. present a large-scale analysis of cell-free DNA (cfDNA) in 12,827 metastatic breast cancer patients. It's a large cohort analysis that revealed the genetic alteration landscape by identifying Guardant360 assay. The manuscript is well written and can serve as an important repertoire for further development, especially given the extent of the data and the number of samples collected. While the manuscript describes the results meaningfully, I have some major concerns:

We thank the reviewer for this positive feedback on the manuscript and our work. Please see our point by point responses below.

Major concerns –
1. The authors should provide a detailed schematic of the samples. Current overview figure given in Figure 1 is not sufficient. It does not describe (in figure or text) the exact time intervals used for collecting the samples. For example, what are the major clinical milestones - which ones are collected before initiating the new therapy, what was the time scale of the disease progression, or the resistance monitoring. Added clarity will help the readers to understand the scale of the experiment.
Thank you for this important feedback. We have added a new panel to Figure 1 (also copied below in point #6) depicting the time of collections between samples from the same patients, which predominantly corresponds to time of progression on treatment. The samples presented had been sent as part of standard of care clinical ctDNA testing via the Guardant 360 commercial platform. For this reason, the timing of testing and the testing intervals were determined by the ordering provider for each individual patient. Given this is a genomically and not clinically annotated dataset, we cannot comment on the specific clinical context of the tests, but rather infer based on clinical practice patterns, including our evolving clinical guidelines. This point is now clarified in the first paragraph of the Results section, and for clinical context, guidelines around next generation sequencing and cfDNA analyses are now clarified in this same paragraph. Today, cfDNA testing is advised at all points of progression for ER+/HER2- breast cancer to evaluate for new *ESR1* mutations. At the time of data collection, general practice patterns typically were to send for testing at diagnosis and at time of progression. We also clarify that on-treatment testing patterns are also captured in this dataset, which is supported by the fact that ctDNA rises have been shown to precede radiographic progression, and thus may inform some practitioners' testing patterns.

2. The demographic information, and age might add another dimension in understanding the factor towards the analysis.

Thank you. The available demographic data obtained through the commercial testing platform, this included patient reported gender and age, which is included in the first paragraph of the results section and Supplemental Table 1.

3. The prediction scores using random forest classifiers are described without specified uncertainty scores. Specifically, TNBC scores are 73% which seems on the lower side. It would be great to judge the performance in comparison with any external database, albeit with a lower number of samples.

Thank you for this comment. We have added the confidence intervals for the classifiers into the text and added error bars to the figures (lines extending from each point). To address the concern about external performance, we have now tested these classifiers on the Metabric dataset and report these results in the supplementary figures (copied also below). The performance of the classifier was highly consistent with the results we obtained on our cohort.

4. The tumor suppressor mutations and oncogenes heatmap (Figure 1d) does not explain the color schemes in the legend. Adding to this maybe stratification of the type of mutation (passenger vs driver) could be interesting to know. Overall the readability of Figure 1d should be improved.
Thank you for this feedback. The heatmap has now been adjusted based on the above guidance.

5. Figure 1e describes the flow of the algorithm. But it would be better to use some notations to denote the variables, the algorithm is predicting and the input feature being used. Then the rest of the discussion about prediction probability becomes clearer.

Thank you for this guidance. We agree. Figure 1e has now been revised accordingly.

6. In the similar direction Figure 1f-h is very hard to comprehend. We understand that allele frequency is related to mutation rate, and there are mutation events related to particular genes. The probability of detection is hardly meaningful and looks like a dark bar. The authors should provide a breakdown of why specific mutations drive classification decisions. Use of log-scale might help with low-frequency mutations. Additionally, any external classification (for example detection by biopsy), would be good to know as a baseline of the prediction. Adding some overall numbers or figures (such as violin plots) would be helpful to understand the distribution of the probabilities across 3 different types of cancers.

Thank you for your suggestions. We have now reworked Figures 1f-h (an example also copied below). In particular, we have: i) changed the mutation rate figure into log-scale; ii) removed the probability tracks; iii) added point distribution plots on the right that show the allele fraction distribution of mutations in specific genes (log-scale); and iv) added a supplemental figure with summary across cohorts for the 3 cancer subtypes.

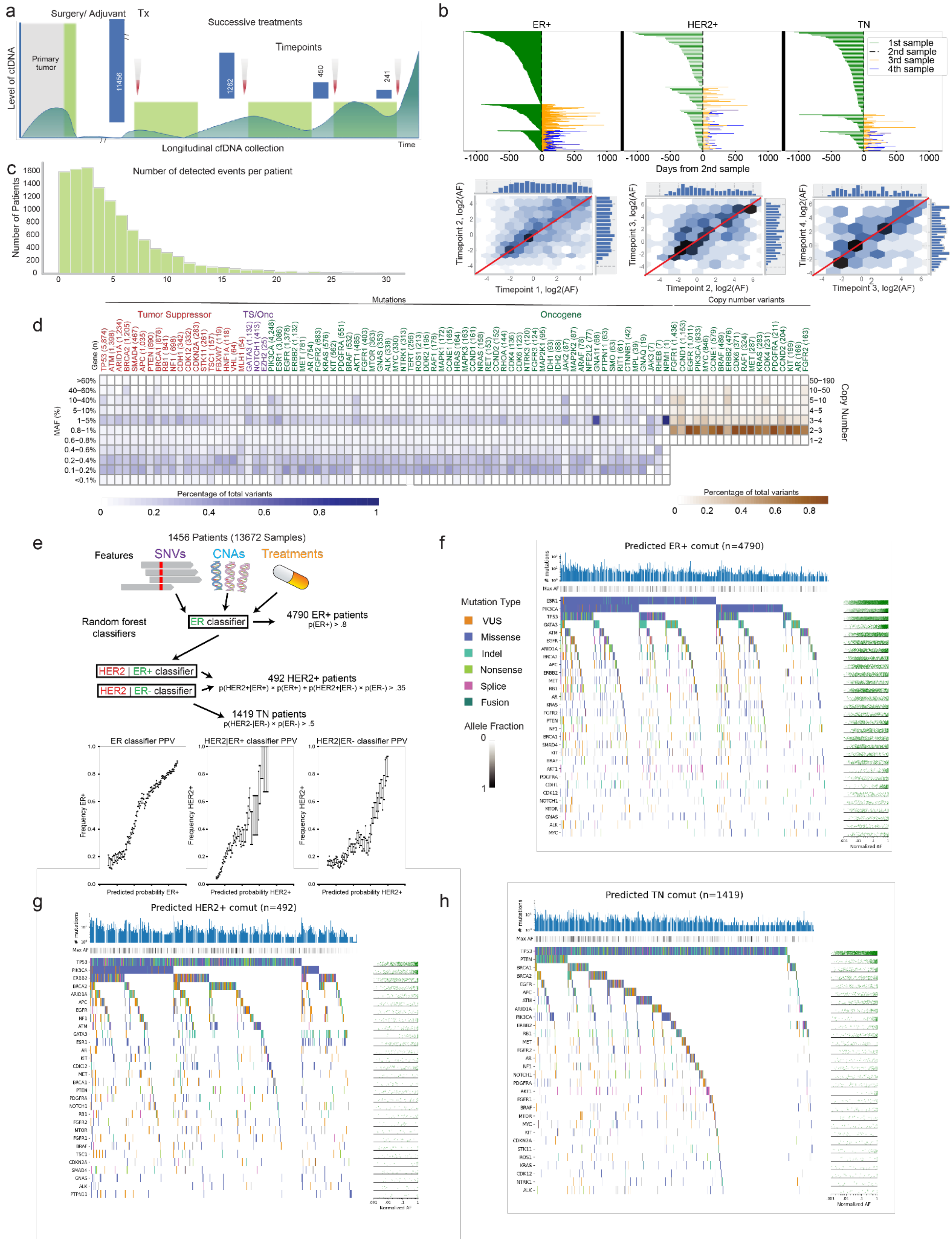
a
Surgery/ Adjuvant
Tx
Successive treatments
Timepoints
Primary tumor
Level of ctDNA
11456
1262
450
241
Longitudinal cfDNA collection
Time
b
ER+
HER2+
TN
1st sample
2nd sample
3rd sample
4th sample
Days from 2nd sample
Timepoint 2, log2(AF)
Timepoint 1, log2(AF)
Timepoint 3, log2(AF)
Timepoint 2, log2(AF)
Timepoint 4, log2(AF)
Timepoint 3, log2(AF)
c
Number of detected events per patient
Number of Patients
d
Mutations
Copy number variants
Tumor Suppressor
TS/Onc
Oncogene
Gene (n)
MAF (%)
Copy Number
Percentage of total variants
Percentage of total variants
e
1456 Patients (13672 Samples)
Features
SNVs
CNAs
Treatments
Random forest classifiers
ER classifier
4790 ER+ patients
p(ER+) > .8
HER2 | ER+ classifier
HER2 | ER- classifier
492 HER2+ patients
p(HER2+|ER+) × p(ER+) + p(HER2+|ER-) × p(ER-) > .35
1419 TN patients
p(HER2-|ER-) × p(ER-) > .5
ER classifier PPV
HER2|ER+ classifier PPV
HER2|ER- classifier PPV
Frequency ER+
Frequency HER2+
Frequency HER2+
Predicted probability ER+
Predicted probability HER2+
Predicted probability HER2+
f
Predicted ER+ comut (n=4790)
Mutation Type
VUS
Missense
Indel
Nonsense
Splice
Fusion
Allele Fraction
Max AF
Normalized AF
g
Predicted HER2+ comut (n=492)
Max AF
Normalized AF
h
Predicted TN comut (n=1419)
Max AF
Normalized AF

7. Figure 2a is very informative in knowing the actionable cancer types. Is it possible to add the longitudinal axis to this. Currently we don't see the time to be a factor of change in the bar plots. Additionally, the relation to the frequency of mutation is not clear to me. For example, are low frequency mutations (rare) less actionable? I am also a little concerned about the class imbalance in the study, for example ER+ patients overwhelm the whole study. Is it possible to sample the dataset to make a balance representation and then evaluate the effects?

Thank you for this suggestion. We have added a longitudinal aspect clarifying this is the percentage of the highest ESCAT level of actionability per patient per subtype. We also completely agree that ER+ patients are disproportionately represented in targetable variants, which is actually due to the fact that more drugs have been studied and approved in this context. This clinical landscape explanation is now clarified in the results section text and addressed as a limitation (and as an argument for more focused genomic studies in non-ER+/HER2- breast cancer) in the discussion. We have now receptor-status-separated versions of Figure 2a in the supplementary figure (S2) shown below.

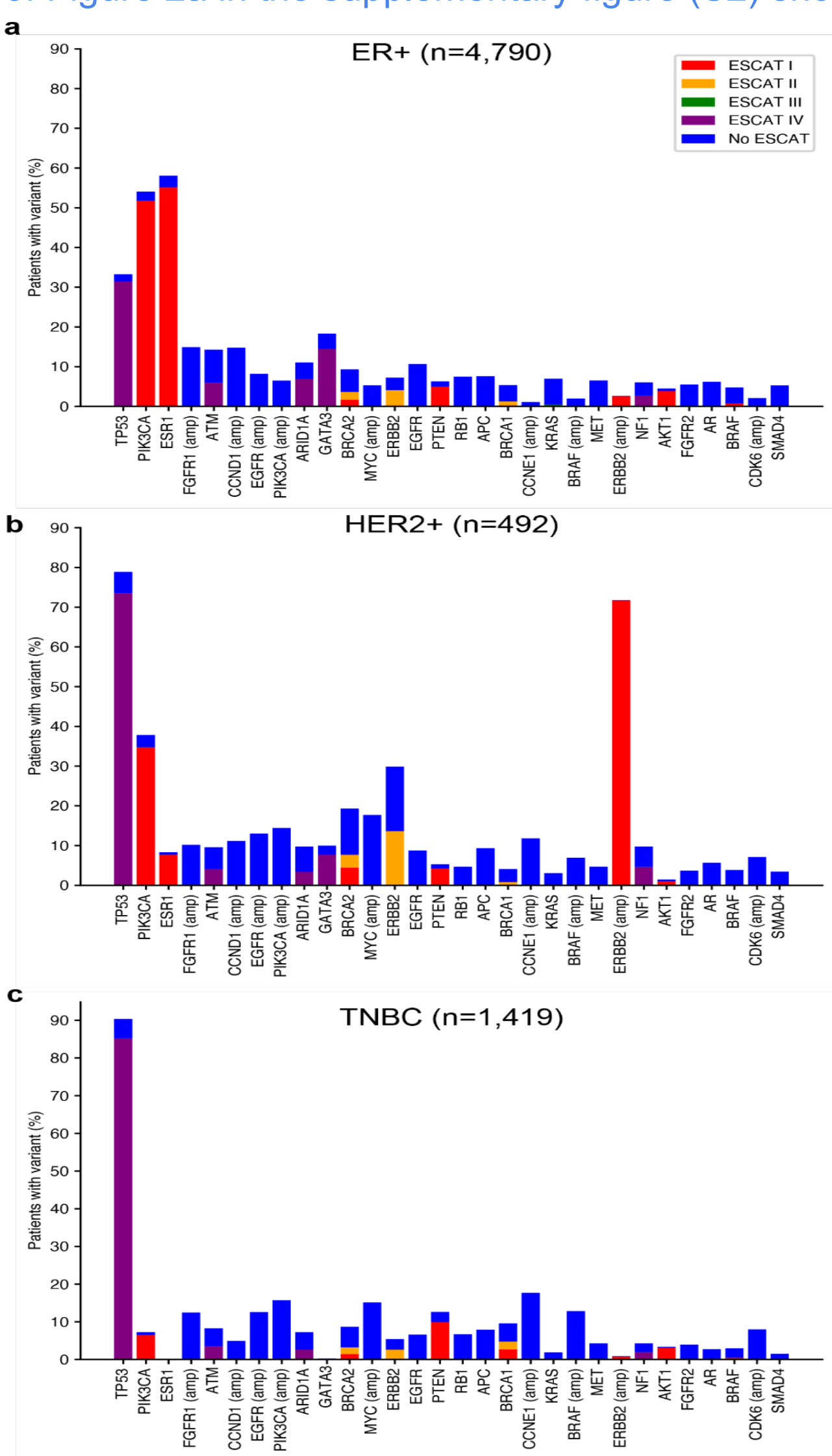

S2

8. Cis vs trans mutations are very important phenomena to study and can have practical impact on tumor-growth and treatment strategy. While figure 3 describes the major observations, I feel the study itself is not well-explored. For example, it is hypothesized that trans-ESR1 mutations have worse therapy outcomes due to independent resistant clones, but no survival analysis is done directly. Moreover, having a parallel copy number analysis can validate this directly at least on some selected patients. Another line of interesting validation could be functional – one a model organism the authors can design CRISPR-engineered breast cancer cell lines harboring single vs. multiple PIK3CA mutations as proxy in same vs different alleles and directly measure the activation of PI3K inhibitors. A differential analysis would directly prove if their hypothesis is valid. Another potential analysis could be checking the transcriptional landscape of cis vs trans mutation.

Thank you for this feedback and suggestions. While this sample set (being a real-life commercial clinical cohort) does not include tissue samples, RNA or survival data, we agree that these findings may inform future studies. This point is now clarified in the discussion. The main noticeable cis mutation for *ESR1* is a di-nucleotide variant that likely happens as a single mutational event. We have performed copy-number analysis on the co-occurrence patterns in the top mutated genes (Methods), which can at least partially explain the prevalence of specific alleles in trans with each other.

9. Clonal occurrence between 67 pairs of mutations is an interesting observation. PIK3CA-GATA3 co-occurrence pattern is unique to MBC samples. Is it possible to validate if this is due to therapy-driven selection. Following my last suggestion, is it possible to do multi-region tumor sequencing from metastatic lesions to determine if the co-mutations occur in distinct clones? This analysis can also be used to actually model co-evolution. Can this be modeled as a joint distribution of mutation where marginal distributions are fixed (e.g. they are Poisson samples etc.)

Expanding on PIK3CA-GATA3 mutation, that co-occurs significantly according to MBC patients – does it also reshape the metabolomics landscape - e.g. shift tumors toward glycolysis? Performing metabolomic analysis could reveal this.

Thank you for this feedback and suggestions. Regarding the clinical rationale for PIK3CA-GATA3 co-occurrences, we have added comments in the text and referenced our recently published clinical work in evaluating the GATA3 mutational landscape in endocrine resistant, metastatic ER+/HER2- breast cancer, which mechanistically supports the observation in our cohort. Regarding the suggestion of a metabolomic analysis and multi region sequencing, this is a very compelling question, which we would be excited to explore in future studies. We would like to point out that this sample set does not include tissue samples (as a clinical blood-biopsy cohort), and while such analysis would not be possible on this specific dataset, we do agree that these findings may inform future studies. This point is now clarified in the discussion.

10. While SERD therapy response is revealing, it relies solely on cell-free DMA to infer the response. It is possible to identify some key samples and do sc-DNA-seq of serial biopsies – this helps in differentiating between pre-existing clones and clones generated due to therapy.

Thank you for this feedback, and we agree that for this reason, these findings are hypothesis generating but not definitive proof of causation. This dataset unfortunately does not include tissue samples that could allow for serial analysis or scDNA-seq outside of the ctDNA domain, as it is a collection of commercial blood biopsy samples from multiple clinical institutions. We thus include this as a limitation. To strengthen the SERD response data, we also have re-analyzed the clinically annotated samples for patients treated at the Mass General Cancer Center and nearly doubled the patient population. Findings remain statistically significant across the entire population, as well as in a genomically-defined *ESR1*-mutated subset, which is now clinically relevant given the approval of oral SERD monotherapy in this genomically-defined subgroup.

11. The survival analysis does not take into account the tumor microenvironment influences at all. While it is not directly part of the study, but could significantly change the clonal selection.

Thank you for calling attention to this important point. We have now clarified this point in the text, and addressed the fact that while we cannot infer immune pressure through cfDNA studies alone, we may use these findings to inform our future clinical studies using solid tumor profiling.

Minor concerns –
- Typo line 392 “increase increase”

Thank you - typo corrected.

- Some sentence are not clearly written – e.g. “as well as further down the PI3K-AKT pathway as AKT inhibitors.” – what does it mean? or “which may suggest the impact that time on treatment".

Thank you for pointing these out - the sentences have now been edited for clarity: “Furthermore, multiple studies are evaluating drugs targeting individual mutations in *PIK3CA*, as well as genes further down the PI3K-AKT pathway (e.g. AKT inhibitors).” and “Given current drug approvals, patients with ER+ disease are more likely to receive a variety of targeted therapies for prolonged periods of time; these targeted standard-of-care therapies include SERDs, aromatase inhibitors, CDK 4/6 inhibitors, PI3K inhibitors, AKT inhibitors, and PARP inhibitors. We hypothesize that prolonged time of targeted therapy may facilitate progression and thus development of multisite resistance as the cancer evolves.”

Overall, I feel this paper will provide useful resources to the community, however it lacks functional validation to back the claims. Complementarily the manuscript also does not make a principled mechanistic model of mutually dependent clonal evolution. The breadth of the

analysis is appealing but it lacks depth (in both method development or building computational models). To make the present manuscript impactful, I strongly believe, the authors should expand on either or both of these directions.

We thank the reviewer for this thoughtful critique. We agree that our study is best positioned as a foundational resource for the community rather than as a definitive mechanistic or experimentally validated model. As noted, there are intrinsic limitations to cfDNA-only datasets that restrict direct functional validation and mechanistic inference. To address this, we have revised the manuscript to more explicitly delineate the scope of this study contributions as providing a comprehensive, well-curated resource and analytic framework to enable hypothesis generation and downstream mechanistic testing by the community. We feel, while the manuscript does not attempt to deliver experimental validation or a fully principled mechanistic model, its strength lies in the breadth and resolution of a real-life clinical data resource establishing a foundation for future mechanistic and functional studies. We have also expanded the Discussion to clarify both the current limitations and the specific opportunities where integration with orthogonal data types and experimental systems will enable deeper mechanistic modeling of clonal dependencies in the future.

Reviewer #2 (Remarks to the Author):

Leshchiner et al presents the largest dataset to date of cfDNA samples from patients with metastatic breast cancer (and possibly with any cancer type), comprising 15,564 samples from 12,827 patients analyzed using the Guardant360 assay. The authors report comprehensive genomic profiling of actionable alterations across breast cancer subtypes, develop a novel cfDNA-based classifier to predict breast cancer subtypes, analyze resistance mechanisms, examine co-occurring mutations, and study clonal dynamics through longitudinal sampling. The work provides valuable insights into the genomic landscape of MBC and has potential implications for clinical trial design and personalized treatment approaches.

We are grateful for the reviewer's positive assessment of our work and manuscript. Please see our point-by-point responses to the comments below.

However, there are several concerns about the manuscript:
Major Concerns
1. The study does not validate whether the detected cfDNA mutations (e.g., ESR1, PIK3CA) are somatic or reflect tumor biology. Germline variants, clonal hematopoiesis (CHIP), or mutations from normal/premalignant tissues (e.g., TP53 in aging) may confound the results. It would be helpful to perform orthogonal validation using matched tumor tissue biopsies to assess concordance between cfDNA and tumor mutations, particularly for targetable variants (e.g., ESR1 ligand-binding domain mutations).

Or if there are any report on the validation Guardant 360 assay (even on other tumor types), the authors can also use the data to give an estimation that what percentage of cfDNA mutations are somatic.

Thank you for raising this issue, which the authors agree with and highlight in the current limitations section of the manuscript. We understand the interest in testing additional biologic specimens to attempt to clarify the origin of each variant call in the study. This dataset unfortunately does not include tissue samples for additional testing (being a real-life commercial clinical cohort). To directly answer the questions about validation, which has been done by Guardant as a very important part of the assay development process: proportion of mutation calls predicted to be clonal hematopoiesis (CH) by various algorithms developed for Guardant and other liquid biopsies range drastically by gene tested, and in healthy donor samples 18% of variants detected in plasma were unable to be identified in WBC sequencing (e.g. Fiaux et al AACR 2025). Additionally, many reports, including by our group, in the literature indicate that paired plasma and single-site tissue testing are not entirely concordant, particularly in cases of pretreated patients as in this report, due to tumor evolution and spatial heterogeneity as different lesions develop different mutations (e.g. Parikh et al Nat Med 2019, Pectasides et al Cancer Discovery 2018, Goyal et al Cancer Discovery 2017), but the specific rate of this discordance being driven by biologic acquired tumor heterogeneity vs contributions from other compartments is, again, variable based on clinical history and biologic context. Gemline variants are clearly seen in patients as having ~50% AF in our data, while most somatic mutations are much lower AF given lower cfDNA tumor fraction. We acknowledge and mention in the discussion that certain genes included in the plasma testing for this study may be more prone to CH confounding (e.g. TP53, ATM, CHEK2) than others (e.g. ESR1, ERBB2), which is noted as a limitation, and we have applied methodology to note mutations in the dataset that are likely CH and germline based on VAF. Furthermore, genes such as *ESR1* and *ERBB2* have hotspot mutations known to be involved in cancer and have not been reported in CH.

2. The assay cannot distinguish germline mutations (e.g., BRCA1/2) or CHIP-derived variants (e.g., TP53, ATM). This is critical for interpreting targetability (e.g., PARP inhibitors require germline/somatic BRCA status). Among these sources of non-somatic mutations, those from CHIP can be filtered by sequencing the WBC DNA. However, the authors didn't perform this analysis, either. I suggest to discuss limitations of relying solely on VAF thresholds (e.g., 40% for BRCA1/2 germline/somatic distinction) without paired germline testing. CHIP-filtering algorithms (e.g., using variant allele frequency [VAF] and gene-specific CHIP patterns) could also be applied for this issue.

Thank you for your suggestion and for calling attention to this important point. These limitations are now additionally highlighted in the discussion. We also discuss the limitation about VAF filtering to identify germline vs somatic origin is an imperfect method, as VAF can be impacted by copy number alterations that could make germline-origin calls appear at VAFs less than 40%, or somatic calls (e.g. in a high tumor burden case) appear at VAFs greater than 40%.

3. The authors should validate the classifier in an independent cohort with pathology-confirmed subtypes. In the current manuscript, the performance of the classifier is based on cross validation, which lead to concerns of overfit. It would be better to use half of cases to train the classifier and use the rest for an independent validation. A weaker validation (if the cases available for this analysis is too limited) is to apply the classifier to the rest cases without label of subtype. And the clinical features of TNBC cases identified by the classifier should be compared to those from TNBC cases from independent cohorts and confirmed by pathology.

Thank you for this suggestion. To address the concern about the classifier performance, we have now tested these classifiers on external Metabric dataset and report these results in the supplementary figures (1a, copied also below). The performance of the classifier was highly consistent with the results we got on our cohort.

a

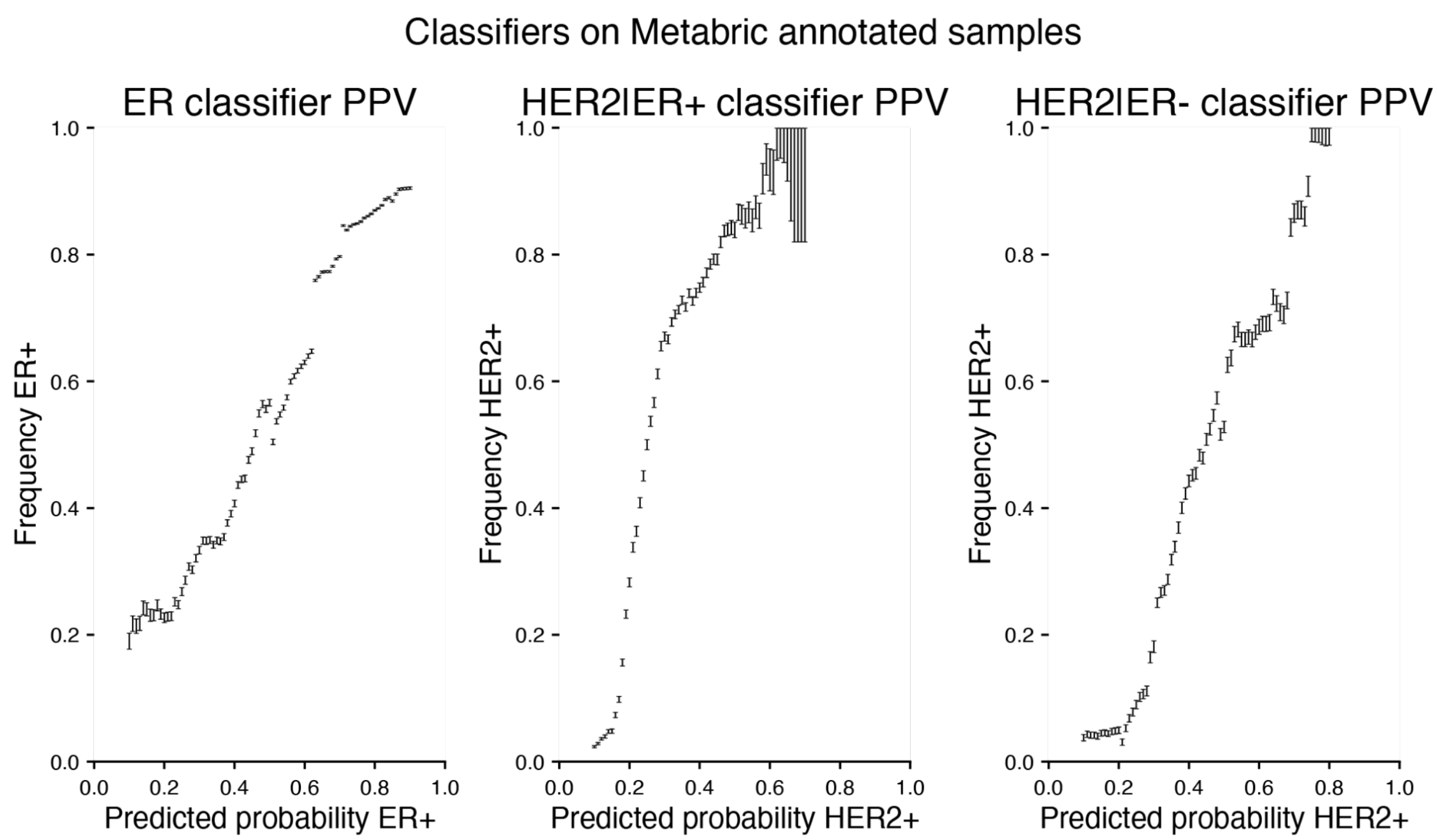

4. I suggest to include more details like cfDNA yield, tumor fraction thresholds, or library preparation QC metrics. Clarify inclusion criteria (e.g., minimum cfDNA concentration, tumor fraction thresholds) and their impact on variant detection.

Thank you for these suggestions. We agree, these details have now been added to the Methods. All samples and results met standard requirements including quality control metrics for clinical testing for CLIA-approved laboratory test, and the methodology for the testing itself, including technical details of analytical process and validity, have been previously published (We have added the citation regarding assay validation via Odegaard et al. This reference contains methodology and figures that demonstrate variant calling performance across levels of input, VAF, etc.).

5. Statistical Thresholds: Using FDR q < 0.1 is overly permissive. Adopt q < 0.05 or justify the lenient threshold with sensitivity analyses.

We added this suggestion thank you.

6. The ESR1 Mutations and ER+ Classification seems circular reasoning: ESR1 mutations are both a classifier feature and a claimed biomarker of ER+ status. Try retraining the classifier excluding ESR1 mutations to evaluate its dependency on this feature.

Thank you for this suggestion. To test the robustness of our classifier we have removed *ESR1* mutations as a feature as well as performed leave-one-out validation for other top genes, the results of the retrained classifier were concordant. We have added the figure below to supplementary figures (**1b**).

b

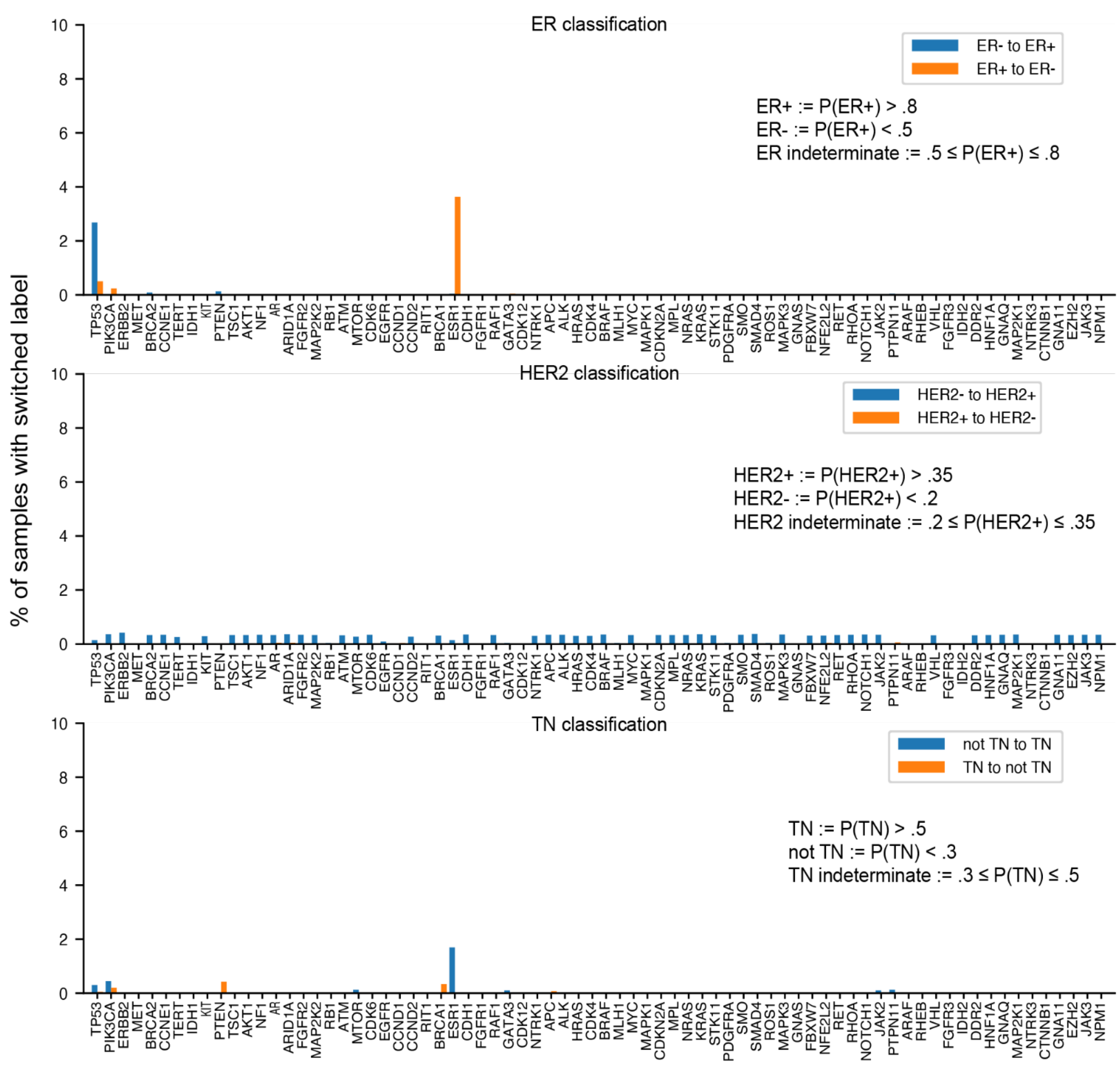

7. The gene pair correlation results are interesting (Fig. 4a). However, the authors should acknowledge the hypothesis-generating nature of these observations and avoid causal language like "synergistic" interactions (e.g., GATA3-ESR1)

Thank you for this point, we have emphasized these findings are hypothesis generating and made sure to not use overly conclusive language.

8. The authors admitted that copy number calls (e.g., FGFR1/CCND1 amplifications) are unreliable in low tumor fraction samples. They should restrict analyses to high tumor fraction samples or validate Guardant360 CNV calls against orthogonal methods (e.g., FISH) or use Guardant data (if any) to show the validation.

Thank you for this feedback. We have referenced Odegaard et al regarding the validation of this assay, including regarding CNA detection performance. Odegaard et al demonstrated high concordance with FISH.

5. The MGH Cohort is too small (n=48). Survival differences (PFS/OS) in SERD-treated patients are underpowered and lack multivariate adjustment (e.g., prior therapies, CDK4/6i use). The authors should try to expand the cohort or contextualize findings as exploratory. A big advantage of this manuscript is the huge size of the cohort. However, if the major/clinically important conclusions are from very small fraction of cases, the power of the huge cohort could be compromised.
Thank you for this feedback. We have now updated the cohort to include almost twice as many patients (n=83) as this initial analysis, and we also did additional analyses for patient subpopulations with detectable *ESR1* mutations given there is now an oral SERD approval for elacestrant for patients with ER+/HER2- advanced breast cancer and detectable *ESR1* mutation(s). The data is much more robust and shows statistically significant differences in progression-free and overall survival across the whole cohort as well as the *ESR1*-mutation subgroups. The text has been updated with these updated results, as well as Figure 5. Additionally, the results of the *ESR1*-mutation cohort have been included as a new supplemental figure. We go further to show show that independent of *ESR1* mutations, the assessment of the genomic landscape at large also correlates with PFS and OS, even when accounting for other factors including age, visceral metastases, prior CDK4/6 inhibitor exposure, and *ESR1* mutation detectability.

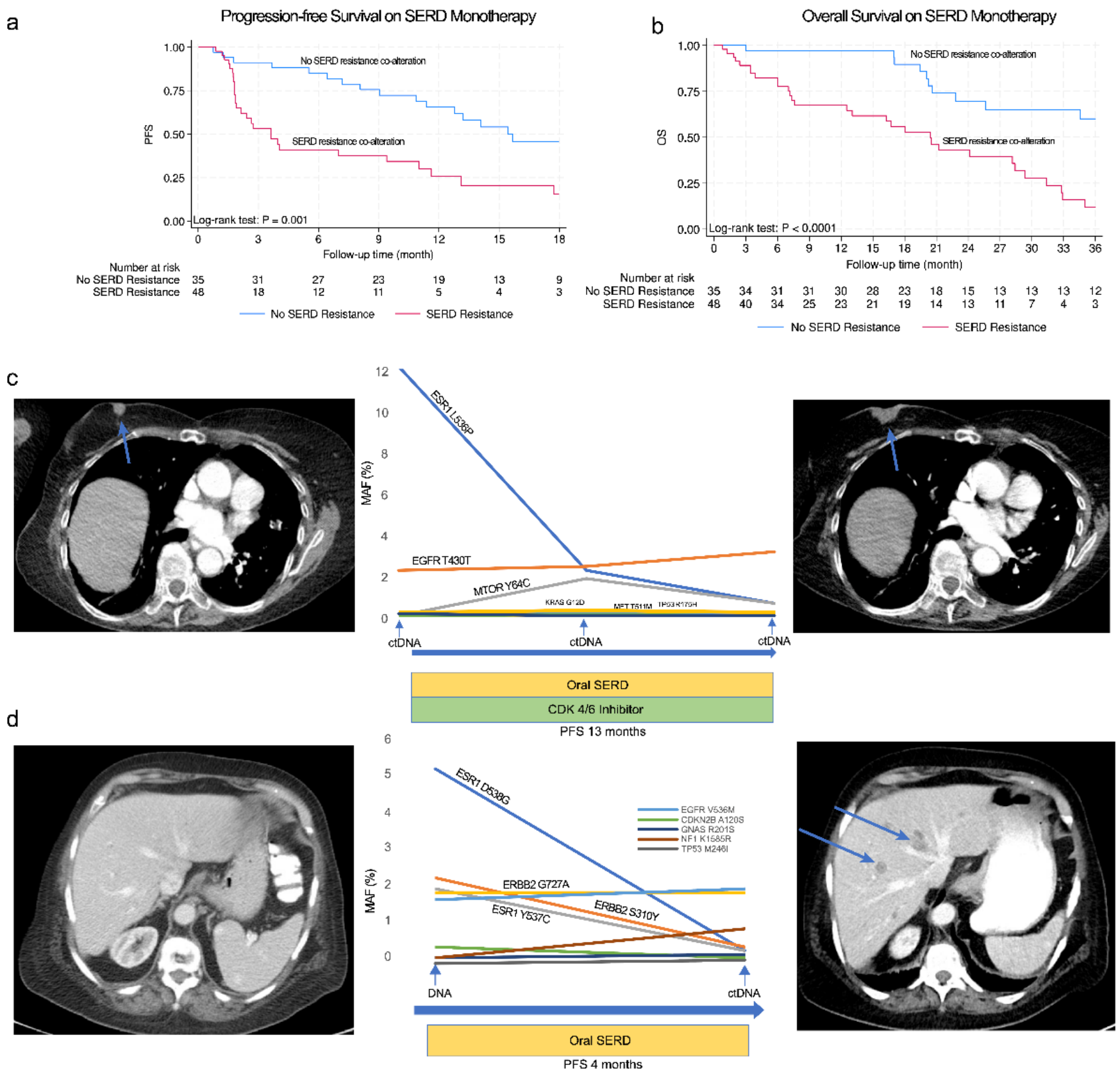

Minor concerns

1. Figure 1d (Genomic Landscape): Overly dense and lacks clarity. Maybe it’s better to split into subpanels (e.g., mutation types, CNVs) and define "TS/Onc" categories.

Thank you for this feedback. The figure has been adjusted according to this feedback. Columns have been added and the definition of TS/Onc has been included in the figure legend.

Figure 3 (Co-occurrence Analysis): Poor resolution in Upset plots; cis/trans phasing lacks statistical rigor.

Thank you for these suggestions. We have improved the resolution of plots in Figure 3. We would like to point out that *ESR1* cis/trans phasing was done on the read level as it is possible with hotspot mutations being in near proximity of each other, we have made a clarification in the Methods section part associated with this figure. The main noticeable cis mutation for *ESR1* is a di-nucleotide variant that likely happens as a single mutational event.

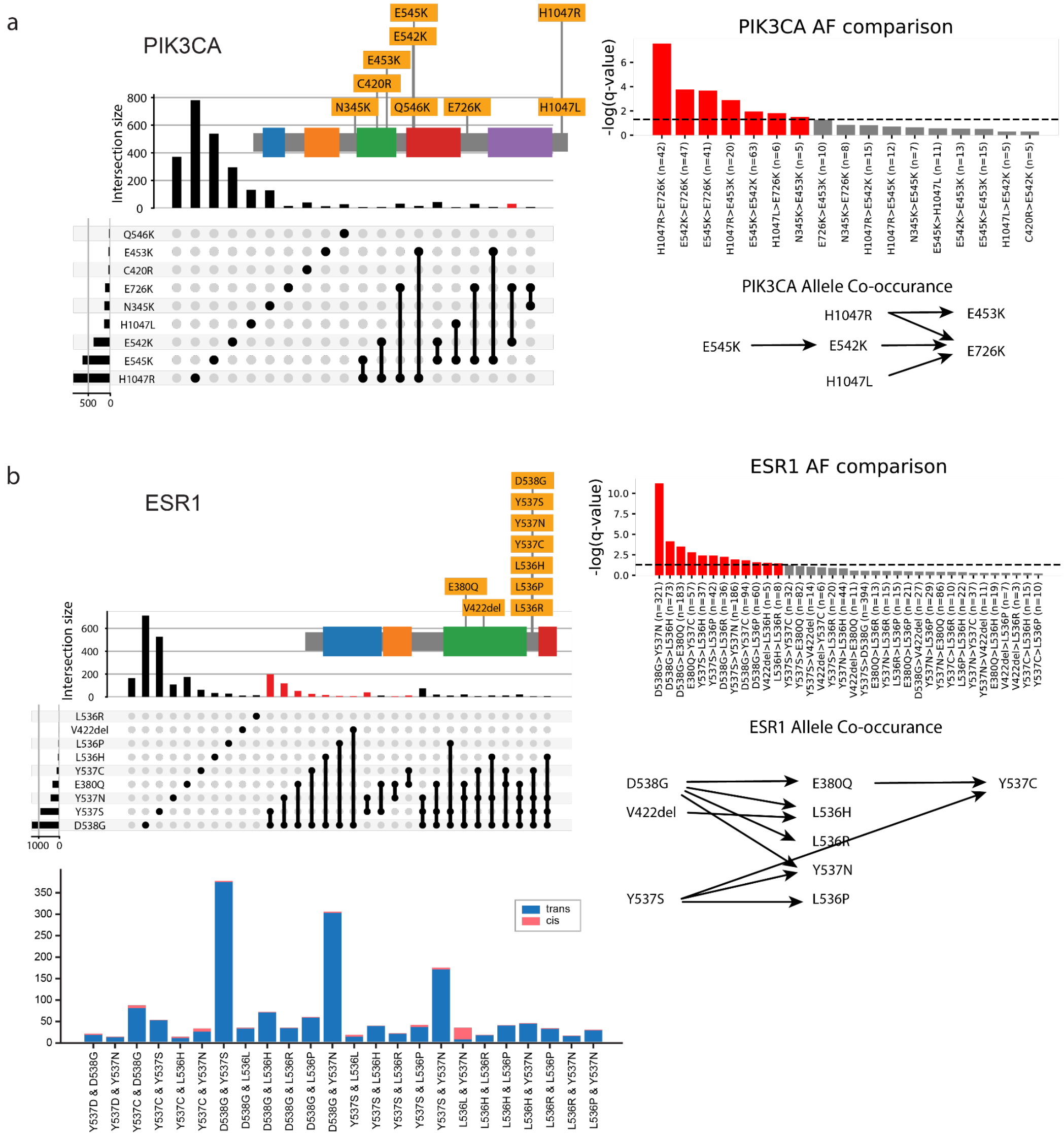

2. Methods Description

Classifier Training:
Feature selection for the Random Forest classifier (e.g., treatment annotations) is unclear.
Thank you for these suggestions. We have now improved the Methods description for feature selection.

Clonal Analysis:
Methods for purity correction (e.g., using "highest AF" as a proxy) and clonal/subclonal categorization are vague.
Thank you for these suggestions. We have now improved the Methods description for clonal/subclonal categorization.

3. It’s better to specify data types (raw FASTQ, processed mutation lists).

Thank you for these suggestions. We have now provided additional descriptions in the Methods.

Reviewer #3 (Remarks to the Author):

The report by Leshchiner et al. characterizes the mutational spectrum of a large cohort of tumors in patients with metastatic breast cancer tested by Guardant using the Guardant360 mutation panel. The sheer size of this cohort is impressive and enables a very comprehensive cataloguing of the most common and most highly coexpressed mutations from amongst those on the panel.
The strengths and novel findings of the study include:
1. The use of a relevant panel of 74 clinicallysignificant genes
2. Depth of sequencing enabling precision
3. Comparison to mutational spectrum in primary tumors from METABRIC and TCGA
4. Careful work looking at co-occurrence of cis and trans mutations, though I am concerned that the "interaction" is not a true interaction but rather correlation - - need to clarify that statistically.
5. Significant correlations between ESR-1 mutations and 6 other genes - - CDH1, CDK6, GATA3, PIK3CA and RHOA. Though the clinical and biological significance of this phenomenon has yet to be elucidated

We are grateful for the reviewer's positive assessment above of our work and manuscript. Please see our point-by-point responses to the comments below.

Despite these strengths, there are numerous issues that limit the generalizeability and utility of the data that is presented.

1. The ctDNA platform does not subtract germline mutations or CHIP. The latter may be particularly problematic in causing overestimation of the mutational burden.

Thank you for highlighting this point. We discuss this issue above in response to Reviewer 2's thoughtful feedback as well. We agree this is a limitation and make a point to emphasize this in the manuscript. This dataset unfortunately does not include tissue samples for additional testing (being a real-life commercial clinical cohort). To directly answer the questions about validation, which has been done by Guardant as a very important part of the assay development process: proportion of calls predicted to be clonal hematopoiesis (CH) by various algorithms developed for Guardant and other liquid biopsies range drastically by gene tested, and in healthy donor samples 18% of variants detected in plasma were unable to be identified in WBC sequencing (e.g. Fiaux et al AACR 2025). Additionally, many reports, including by our group, in the literature indicate that paired plasma and single-site tissue testing are not entirely concordant, particularly in cases of pretreated patients as in this report, due to tumor evolution and spatial heterogeneity as different lesions develop different mutations (e.g. Parikh et al Nat Med 2019, Pectasides et al Cancer Discovery 2018, Goyal et al Cancer Discovery 2017), but the specific rate of this discordance being driven by biologic acquired tumor heterogeneity vs contributions from other

compartments is, again, variable based on clinical history and biologic context. Gemline variants are clearly seen in patients as having ~50% AF in our cfDNA data, while most somatic mutations are much lower AF given lower cfDNA tumor fraction. We acknowledge and mention in the discussion that certain genes included in the plasma testing for this study may be more prone to CH confounding (e.g. TP53, ATM, CHEK2) than others (e.g. ESR1, ERBB2), which is noted as a limitation, and we have applied methodology to note mutations in the dataset that are likely CH and germline based on VAF. Genes such as *ESR1* and *ERBB2* also have hotspot mutations known to be involved in cancer and have not been reported in CH.

2. The data is, for the most part, descriptive, and largely confirms prior findings in albeit smaller datasets.

Thank you for this feedback. We agree that the primary strength of our work lies in the unprecedented size of the dataset, encompassing disease across multiple nationwide centers and patient populations, which enables large-scale analysis and wider clinical context rather than more focused clinically narrow studies. We have now emphasized these limitations more clearly in the text to better frame the scope and impact of the study. To add clinical depth where possible, we expanded Figure 5 to include a clinically annotated cohort of patients treated at the Mass General Cancer Center, focusing on outcomes with SERD monotherapy. As SERDs represent one of the newest targeted therapy classes in breast cancer and have directly influenced guideline recommendations for genomic testing at progression in ER+/HER2- disease, we believe this addition provides valuable clinical context to complement the broader descriptive analyses.

3. The clinical characteristics of patients in the cohort is not described, nor does it seem available for the analysis. Presumably, this is a cohort of patients selected by their clinicians to have the Guardant360 test performed. Given the timeframe in which the cohort data was collected, starting in 2017, this was a very early timepoint during which guidelines did not recommend mutation testing, so a very select group would have had this done. The possible bias is seen in the extremely high frequency of samples harboring at least one mutation Thus there is inherent selection bias – the extent to which this skews the data from what would be seen in an unselected “all comers” type of study is not clear.

Thank you for this feedback. This is indeed true and requires clear context, which has now been added into the limitations of this manuscript to underline this important point and clinical context. We explain that testing may have been set at initial diagnosis, on treatment, or (in most circumstances) at time of progression to assess for mechanisms of therapy resistance and evaluate for genomic therapy targets. Today, cfDNA testing is advised at all points of progression for ER+/HER2- breast cancer to evaluate for new *ESR1* mutations. At the time of data collection, general practice patterns typically were to send for testing at diagnosis and time of progression. We also clarify on-treatment testing patterns are also captured in this dataset, which is supported by the fact that ctDNA rises have been shown to precede radiographic progression, and thus may inform some practitioners’ testing patterns.

4. Moreover the cohort lacks clinical annotation, and thus we are not able to interpret the development of mutations in the context of clinical extent of disease, type of therapy (both over the course of disease and immediately prior to the test) or line of therapy, all of which would be helpful in understanding what the mutational spectrum is telling us about both intrinsic and acquired resistance.

Thank you for this feedback. While the available annotations include therapies as reported by the physician, we agree that such reported annotations are indeed a limitation, where the breadth of the genomic data is the major advantage, and the lack of depth of clinical annotation is a limitation. Contextualization is required and has now been added to the discussion. The large volume of data is very meaningful for understanding the overall metastatic breast cancer genomic landscape, which is increasingly relevant as more and more targeted therapies are developed and approved. We have also now included a deeper analysis into the clinical data of patients treated at the Mass General Cancer Center, which has extensive curated annotations, where we focus on SERD monotherapy and the larger context of genomic data. As more studies around ctDNA-guided therapy decisions are underway, it will be critical to consider the mutational landscape at large. We now highlight practice changing studies such as the approval of elacestrant for *ESR1*-mutations advanced ER+/HER2- breast cancer, and the fact that this approval has led to society guidelines for ctDNA testing in this breast cancer subtype (the most common at 70-80% of cases) at every progression to look for *ESR1* mutations. We also highlight the point that *ESR1* mutations are only a surrogate marker for continued ESR1 dependence, and the larger genomic landscape should be understood in overall context. We also have now added data around the results of SERENA-6 and PADA-1, both of which have shown clinical benefit to early changes in endocrine therapy at first detection of an ESR1 mutation. SERENA-6 employed ctDNA testing every 2-3 months (whereas standard of care testing at the time of progression means patients are having testing closer to every 2 years), thus if these studies were to change the standard of care, we would expect genomic testing to markedly increase in metastatic breast cancer. For these reasons, we feel, this dataset is even more important to understand the broader genomic landscape at large.

5. The small subcohort with clinical annotation appear to be all derived from a clinical trial of elacestrant, which is an even more highly selected group of patients. This data, while interesting, does not seem appropriately included in this manuscript and would be better placed in a separate report as its relevance to the questions being answered in the larger cohort is unclear.

Thank you for this feedback. In order to address the above concerns about clinical annotation, we have performed a new analysis on a larger cohort and made the clinical context clearer in our study description. This was done specifically because of the limitation of the wider dataset wherein there is no deep clinical annotation. We focus on SERD monotherapy to connect our findings to the more recently improved genomically-annotated drugs. This analysis was restructured and re-analyzed to focus both on patients with *ESR1* mutations and the

ER+/HER2- population at large, given the concern that *ESR1* mutations are surrogate markers for ER dependence (and hence vulnerability to SERD therapy) but are imperfect metrics. Here, we show that independent of *ESR1* mutations, co-mutations associated with SERD resistance were associated with worse PFS and OS, even when accounting for other factors including age, visceral metastases, prior CDK4/6 inhibitor exposure, and *ESR1* mutation detectability.

6. There are numerous portions of the manuscript that describe potential uses for ctDNA in metastatic breast cancer, recommendations from guidelines committees including ASCO, and other editorial type comments that do not seem to be relevant to the data being presented.

Thank you for this feedback. We would like to clarify that we provide further discussion about the clinical context for why these results are clinically significant. We envision this tool being used for understanding recruitment opportunities and genomic patterns for future genomic-driven clinical trials by physicians and attempt to clarify this dataset offers itself as a significant resource in both genomic analyses as well as in clinical trial planning to improve future patient care. Also, given the recent approval of the PI3K inhibitor inavolisib, this has been added to the text as well to further strengthen the clinical context.

7. It is not possible to directly measure tumor evolution under pressure of treatment since treatment information is not available. There are sentences (eg 308-309) suggesting this without having any data to back it up.

Thank you for this feedback and suggestion. We have now adjusted the language to be more balanced and avoid any confusion about the interpretation of overall treatment response.

8. Its difficult to understand the clinical value of the ER/HER2 predictor. The vast majority of patients, at diagnosis of metastatic disease, have a tumor biopsy in which receptors are measured. This is used for treatment purposes. The predictor developed by the authors has only 75% accuracy, which is insufficient to replace a biopsy. Moreover, when tumors become resistant to therapies that are targeting these receptors, the receptor expression doesn't change, just the function changes. Thus, a predictor of sensitivity to ER or HER2-directed therapy would be useful; simply predicting the subtype is unlikely to have particular utility in improving outcome.

Thank you for this feedback. We would like to clarify that the main purpose of the classifier in this study is to be able to assign breast cancer subtypes based solely on available data in real-life commercial cfDNA tests, which enables us to calculate frequencies and correlations of events within each subtype across a much larger cohort. We absolutely agree that clinical receptor status is going to be continued being assessed based on tissue biopsy profiling. We have now tested the classifier on external well annotated Metabric dataset and the performance of the classifier was highly consistent with the results we got on our cohort, we added changes to the manuscript to highlight this aspect.

Point by point Response

We thank the reviewers for additional constructive critique of our manuscript.

Reviewer #1 (Remarks to the Author):

The authors have satisfactorily responded to the comments during the first round of review, improved the manuscript and better positioned it as a resource paper.

We thank the reviewer for their time and all the feedback on the manuscript and our work.

Reviewer #2 (Remarks to the Author):

The authors have made substantial improvements, but some critical issues remain:
1.Germline vs. Somatic / CHIP Distinction: The authors mentioned that 18% of plasma mutations cannot be identified in WBC sequencing. Paired plasma-tissue discordance exists due to heterogenenity. These partially explained the issue, but cannot solve the problem. This remains the most critical weakness. We still don't know, what proportion of TP53 mutations might be CH-derived? Etc. This needs additional analysis or at minimum, much stronger language in the Discussion about how CH/germline contamination may inflate certain mutation frequencies and limit clinical interpretation.

We thank the reviewer for their time and all the feedback on the manuscript and our work. Please see our point-by-point responses below.

I would suggest:
(1) Add Supplementary Figure showing VAF distributions for commonly mutated genes;

Thank you for this suggestion. We have now added Supplemental Figure S4a with these distributions.

(2) Perform sensitivity analysis excluding CH-prone genes (TP53, ATM, BRCA1/2, CHEK2);

Thank you for this suggestion. We have performed this analysis and now added Supplemental Figure S4b-d with exclusion of CH genes.

(3) Re-calculate actionability rates with/without these genes;

Thank you for this suggestion. We The contribution of BRCA1/2 variants, which are potential CH variants and also classified as ESCAT I/II is now addressed with exact quantification in the discussion, addressing the limitations.

(4) Add prominent Discussion paragraph acknowledging how CH may inflate certain mutation frequencies

Thank you for this suggestion. In the new Discussion paragraph, where limitations are discussed, we have now included a clear statement about the limitations of the lack of CH distinction, and we specifically highlight BRCA1/2 variants, as described above, in recognition of this being a potential CH variant.

2.Clinical Context/Selection Bias:

a)Add to Discussion: Clear statement that cohort represents selected patients (high tumor burden, high cfDNA yield, which could include more progressive disease)

Thank you for this suggestion. This statement is now clearly made in the beginning of the Discussion.

b)Compare cohort characteristics to published MBC populations
Thank you, this is addressed in further depth now in the third paragraph of the Discussion.

c)Acknowledge generalizability limitations more prominently
This limitation is now more clearly acknowledged in the beginning of the Discussion.

3.Language:
a)Change "interaction" to "correlation/co-occurrence"

Thank you for this feedback. This language has been changed and clarified throughout the manuscript, and any references to interaction have been clarified as solely suppositive based on correlation/co-occurrence in the data.

b) Add "hypothesis-generating" qualifications to correlation findings

Thank you for this feedback. In addition to softening the interaction language above, when we address correlation data in the discussion, we clarify these findings are hypothesis generating.
