# Supplementary material for: Actionable genetic landscape and dynamics in circulating DNA from 12,827 patients with metastatic breast cancer
Source: Nat Commun. 2026 Sep 22;17:11023. doi: 10.1038/s41467-026-75976-9 (PMC13687546; doi:10.1038/s41467-026-75976-9)
Supplement: Supplementary file 2 [file 41467_2026_75976_MOESM1_ESM.pdf]

Supplementary Information and Figures

Supplementary figure 1: Orthogonal classifier validation

a) PPV plots similar to what is shown in **Figure 1e** for annotated METABRIC samples.
b) Differences in results on ER classification (top), HER2 classification (middle), and TNBC classification (bottom) of retrained holdout classifiers on original data vs the full classifier. Each holdout classifier leaves the point mutations of the labelled gene out of the input features. Each pair of bars represents the percentage of patients that switch labels to/from the cohort with thresholds given for each.

Supplementary figure 2: Rebalanced cohort analysis

a-c) Bar plot of mutational frequencies in predicted ER+, HER2+, and TNBC patients, color-coded by ESCAT actionability similar to **Figure 2a**.

Supplementary figure 3: Additional gene mutation frequency comparisons

a) Bar plot comparing mutational frequencies of genes in HER2+ patients to the METABRIC cohort, similar to what is shown in **Figure 2b-e**.
b) Bar plot comparing mutational frequencies of genes in TNBC patients to the METABRIC cohort, similar to what is shown in **Figure 2b-e**.

Supplementary figure 4: Variant allele fraction distributions and classifier validation

a) Variant allele fraction distributions for most frequently mutated genes in the cohort
b) c), d) Positive Predictive Value for individual subtypes of breast cancer samples

Supplementary figure 5: Survival analysis for ESR1-mutated patients

a-b) Overall survival and progression-free survival stratified by presence of co-occurring SERD resistance mutations similar to **Figure 5a-b** but on the subset of ESR1-mutated patients.

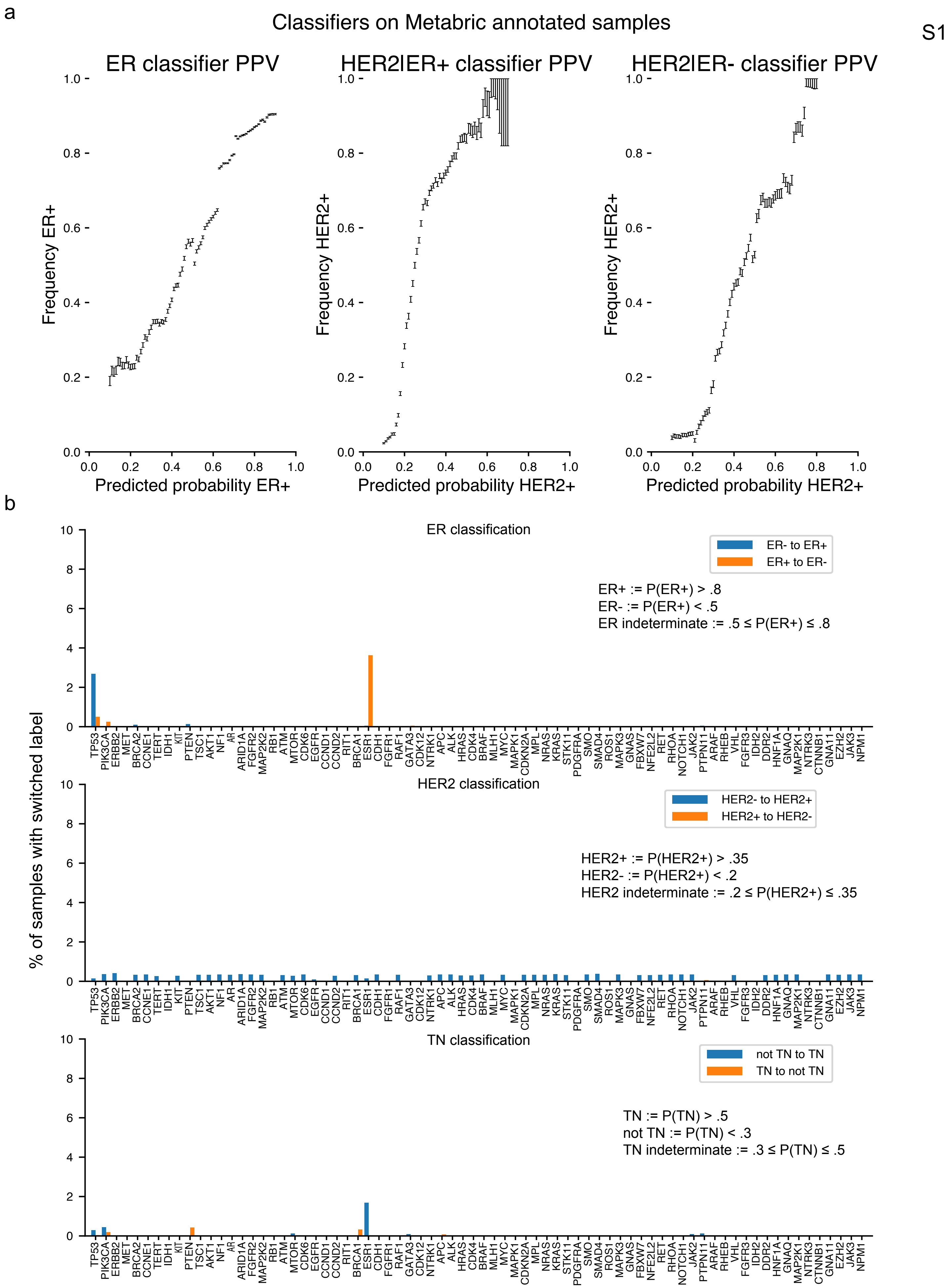
S1
a
Classifiers on Metabric annotated samples
ER classifier PPV
Frequency ER+
Predicted probability ER+
HER2IER+ classifier PPV
Frequency HER2+
Predicted probability HER2+
HER2IER- classifier PPV
Frequency HER2+
Predicted probability HER2+
b
% of samples with switched label
ER classification
ER- to ER+
ER+ to ER-
ER+ := P(ER+) > .8
ER- := P(ER+) < .5
ER indeterminate := .5 ≤ P(ER+) ≤ .8
HER2 classification
HER2- to HER2+
HER2+ to HER2-
HER2+ := P(HER2+) > .35
HER2- := P(HER2+) < .2
HER2 indeterminate := .2 ≤ P(HER2+) ≤ .35
TN classification
not TN to TN
TN to not TN
TN := P(TN) > .5
not TN := P(TN) < .3
TN indeterminate := .3 ≤ P(TN) ≤ .5
TP53 PIK3CA ERBB2 MET BRCA2 CCNE1 TERT IDH1 KIT PTEN TSC1 AKT1 NF1 AR ARID1A FGFR2 MAP2K2 RB1 ATM MTOR CDK6 EGFR CCND1 CCND2 RIT1 BRCA1 ESR1 CDH1 FGFR1 RAF1 GATA3 CDK12 NTRK1 APC ALK HRAS CDK4 BRAF MLH1 MYC MAPK1 CDKN2A MPL NRAS KRAS STK11 PDGFRA SMO SMAD4 ROS1 MAPK3 GNAS FBXW7 NFE2L2 RET RHOA NOTCH1 JAK2 PTPN11 ARAF RHEB VHL FGFR3 IDH2 DDR2 HNF1A GNAQ MAP2K1 NTRK3 CTNNB1 GNA11 EZH2 JAK3 NPM1

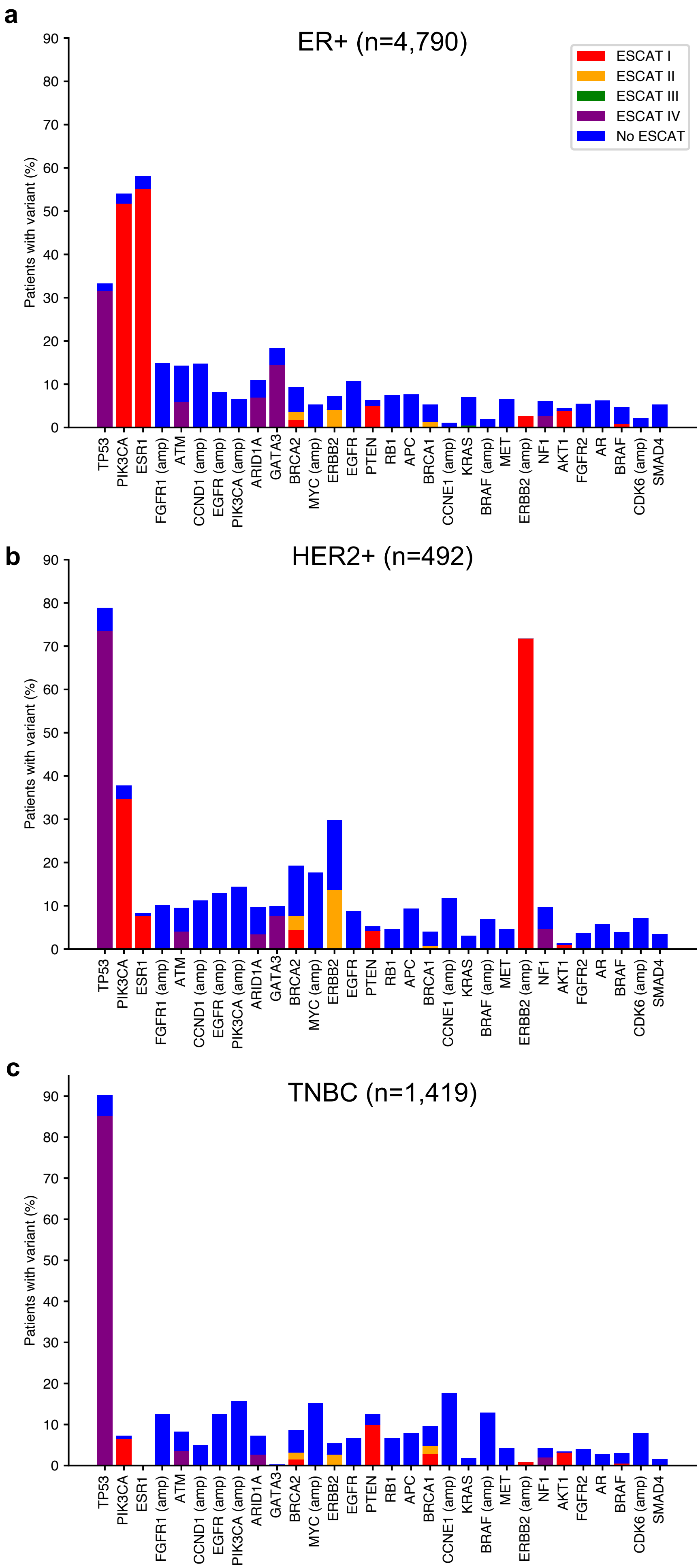
a
ER+ (n=4,790)
b
HER2+ (n=492)
c
TNBC (n=1,419)
ESCAT I
ESCAT II
ESCAT III
ESCAT IV
No ESCAT
Patients with variant (%)
0
10
20
30
40
50
60
70
80
90
TP53
PIK3CA
ESR1
FGFR1 (amp)
ATM
CCND1 (amp)
EGFR (amp)
PIK3CA (amp)
ARID1A
GATA3
BRCA2
MYC (amp)
ERBB2
EGFR
PTEN
RB1
APC
BRCA1
CCNE1 (amp)
KRAS
BRAF (amp)
MET
ERBB2 (amp)
NF1
AKT1
FGFR2
AR
BRAF
CDK6 (amp)
SMAD4

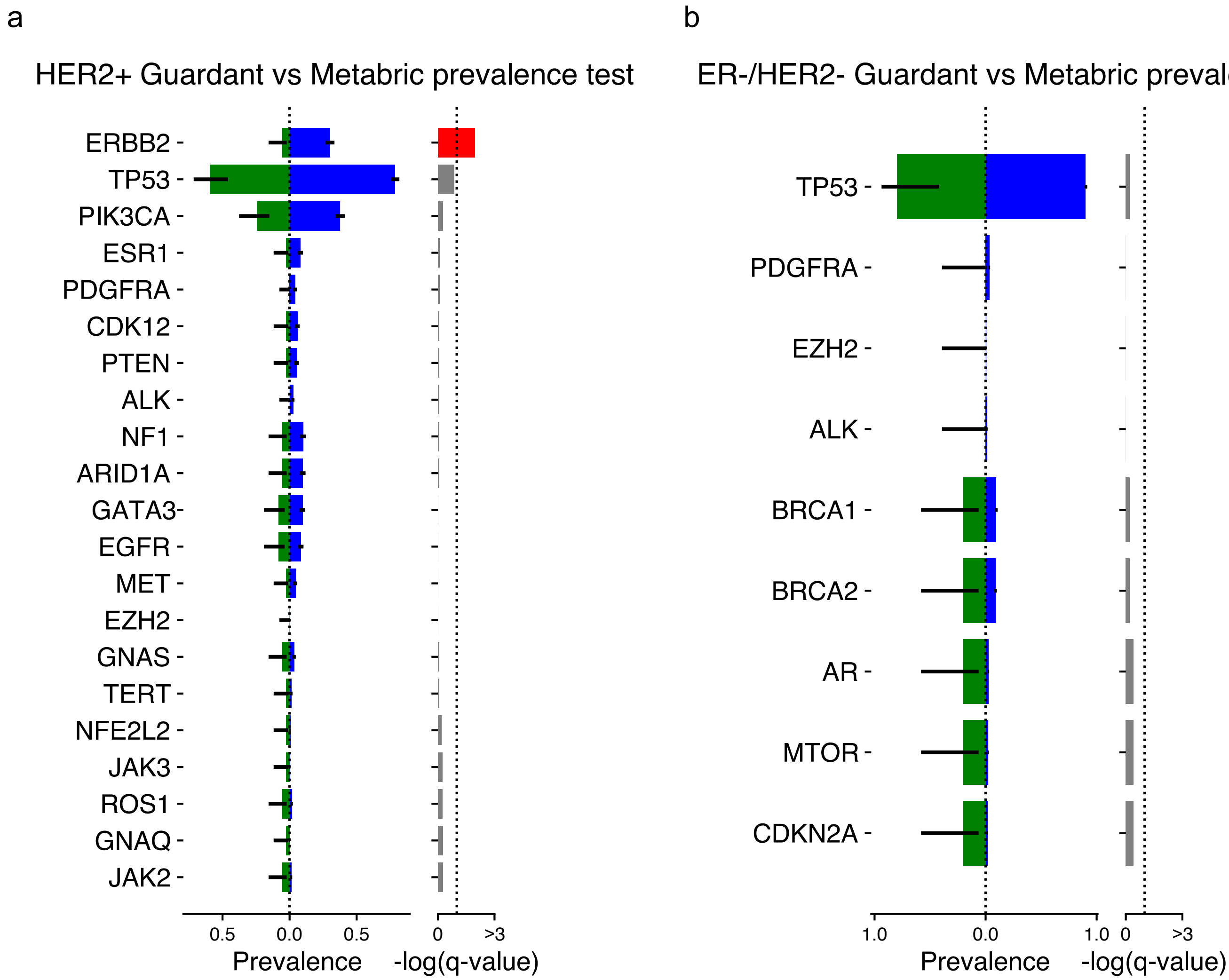
a
HER2+ Guardant vs Metabric prevalence test
ERBB2
TP53
PIK3CA
ESR1
PDGFRA
CDK12
PTEN
ALK
NF1
ARID1A
GATA3
EGFR
MET
EZH2
GNAS
TERT
NFE2L2
JAK3
ROS1
GNAQ
JAK2
0.5
0.0
0.5
0
>3
Prevalence
-log(q-value)
b
ER-/HER2- Guardant vs Metabric prevalence test
TP53
PDGFRA
EZH2
ALK
BRCA1
BRCA2
AR
MTOR
CDKN2A
1.0
0.0
1.0
0
>3
Prevalence
-log(q-value)

S4

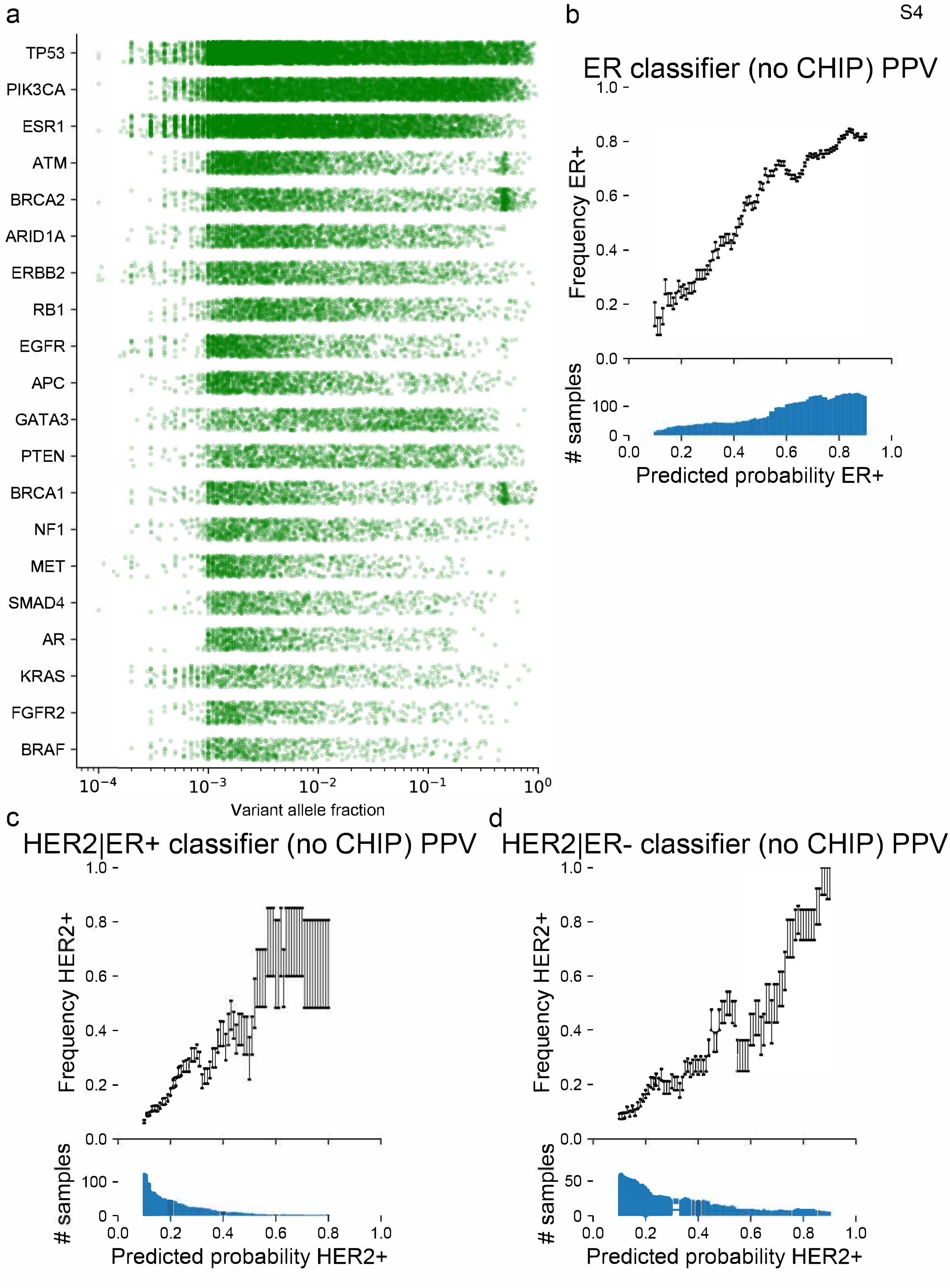
a
TP53
PIK3CA
ESR1
ATM
BRCA2
ARID1A
ERBB2
RB1
EGFR
APC
GATA3
PTEN
BRCA1
NF1
MET
SMAD4
AR
KRAS
FGFR2
BRAF
10^{-4} 10^{-3} 10^{-2} 10^{-1} 10^{0}
Variant allele fraction
b
ER classifier (no CHIP) PPV
Frequency ER+
samples
Predicted probability ER+
c
HER2|ER+ classifier (no CHIP) PPV
Frequency HER2+
samples
Predicted probability HER2+
d
HER2|ER- classifier (no CHIP) PPV
Frequency HER2+
samples
Predicted probability HER2+

a

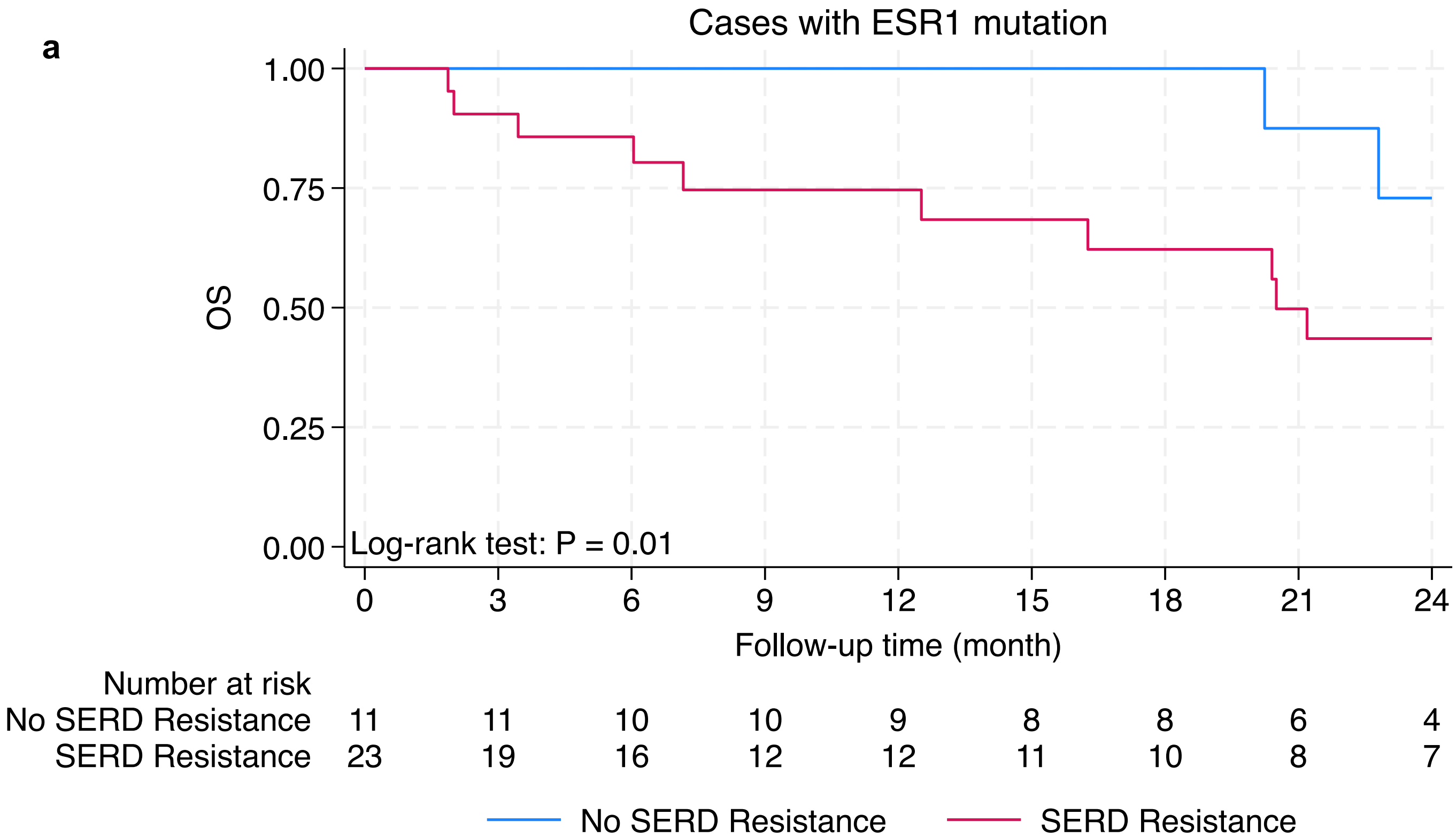
Cases with ESR1 mutation
OS
1.00
0.75
0.50
0.25
0.00
Log-rank test: P = 0.01
0 3 6 9 12 15 18 21 24
Follow-up time (month)
Number at risk
No SERD Resistance 11 11 10 10 9 8 8 6 4
SERD Resistance 23 19 16 12 12 11 10 8 7
No SERD Resistance
SERD Resistance

b

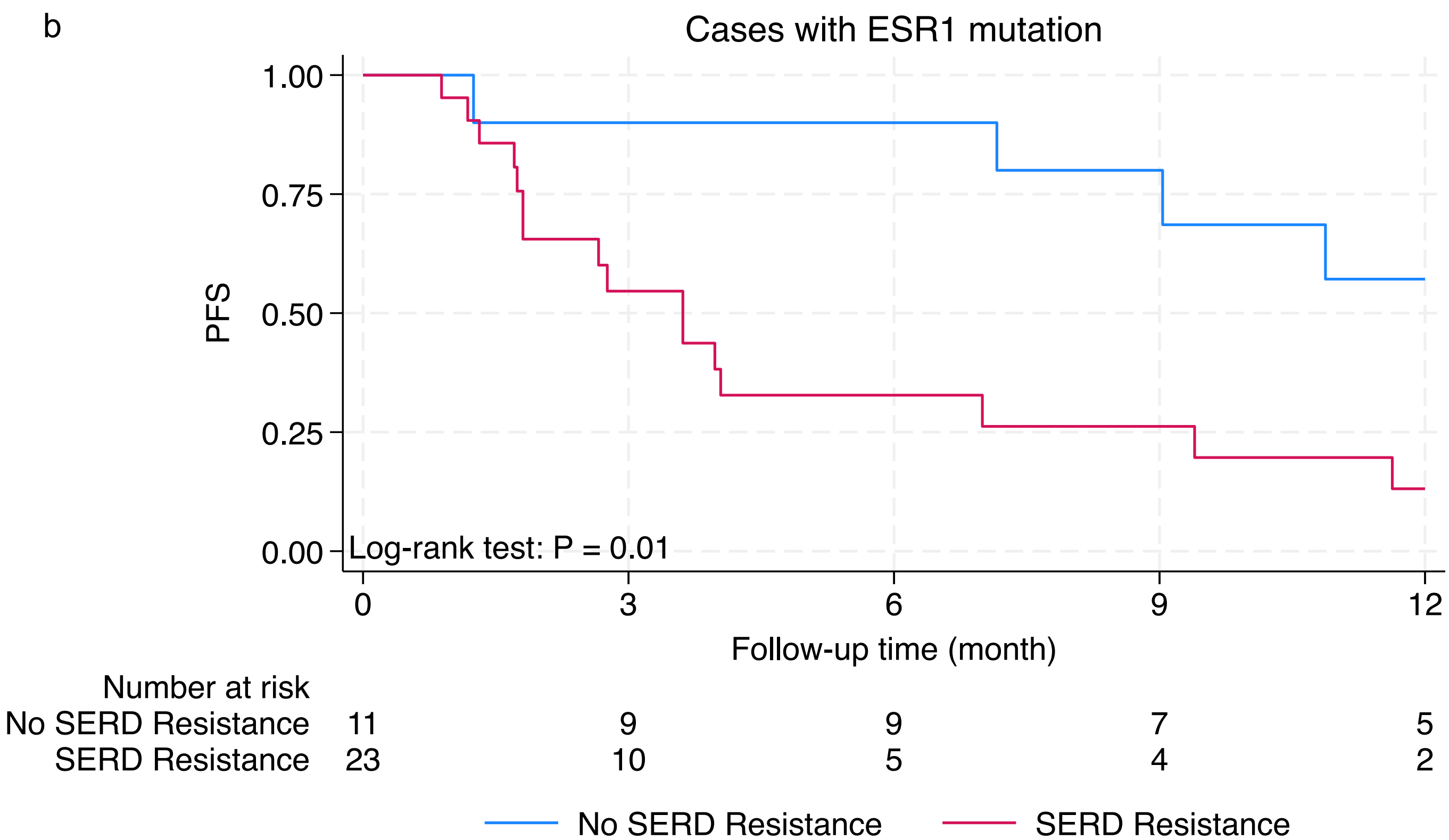
Cases with ESR1 mutation
PFS
1.00
0.75
0.50
0.25
0.00
Log-rank test: P = 0.01
0 3 6 9 12
Follow-up time (month)
Number at risk
No SERD Resistance 11 9 9 7 5
SERD Resistance 23 10 5 4 2
No SERD Resistance
SERD Resistance
