# Supplementary material for: Actionable genetic landscape and dynamics in circulating DNA from 12,827 patients with metastatic breast cancer
Source: Nat Commun. 2026 Sep 22;17:11023. doi: 10.1038/s41467-026-75976-9 (PMC13687546; doi:10.1038/s41467-026-75976-9)
Supplement: Supplementary file 3 [file 41467_2026_75976_MOESM2_ESM.pdf]

Description of Additional Supplementary Files

Supplementary Data 1

Description: The list of genes covered by the Guardant360 panel.

Supplementary Data 2

Description: The full dataset of Guardant360 assay from 15,564 samples.

Supplementary Data 3

Description: Breast cancer subtypes annotated in the dataset.

Supplementary Data 4

Description: Data used in the training of cancer subtype classifier.

Supplementary Data 5

Description: Patients with genomic alterations identified at any point via cfDNA testing.

Supplementary Data 6

Description: Clinical data for SERD Monotherapy treated patients.
